# Supplementary figures and images for: Comparative Transcriptome Analysis Reveals the Molecular Basis of Brassica napus in Response to Aphid Stress
Source: Plants (Basel). 2023 Aug 3;12(15):2855. doi: 10.3390/plants12152855 (PMC10421284; doi:10.3390/plants12152855)

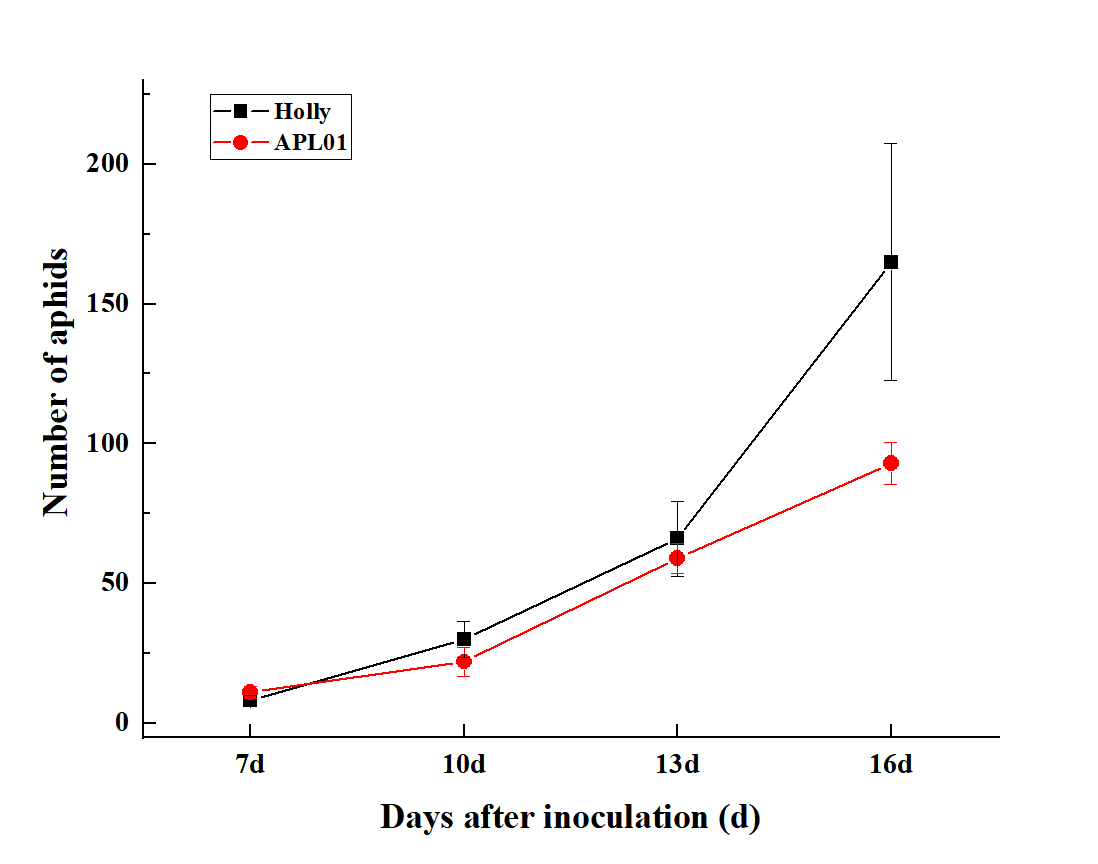

Supplement: Supplementary file 1 [file plants-12-02855-s001.zip › plants-2467175-supplementary/Supplementary materials/Supplementary Figures/Figure S1.tiff]

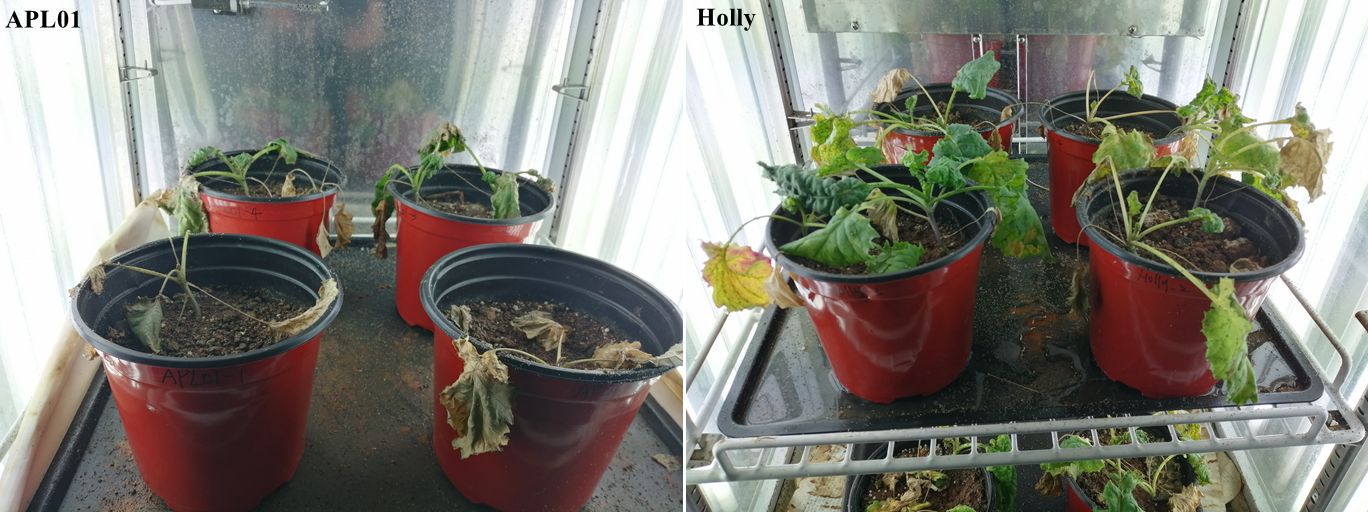

Supplement: Supplementary file 1 [file plants-12-02855-s001.zip › plants-2467175-supplementary/Supplementary materials/Supplementary Figures/Figure S2.tiff]

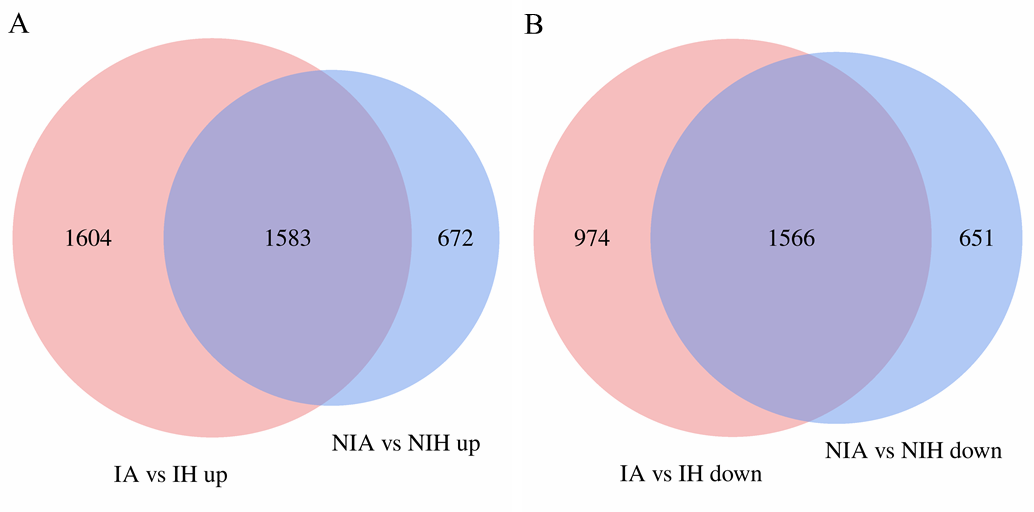

Supplement: Supplementary file 1 [file plants-12-02855-s001.zip › plants-2467175-supplementary/Supplementary materials/Supplementary Figures/Figure S3.tiff]

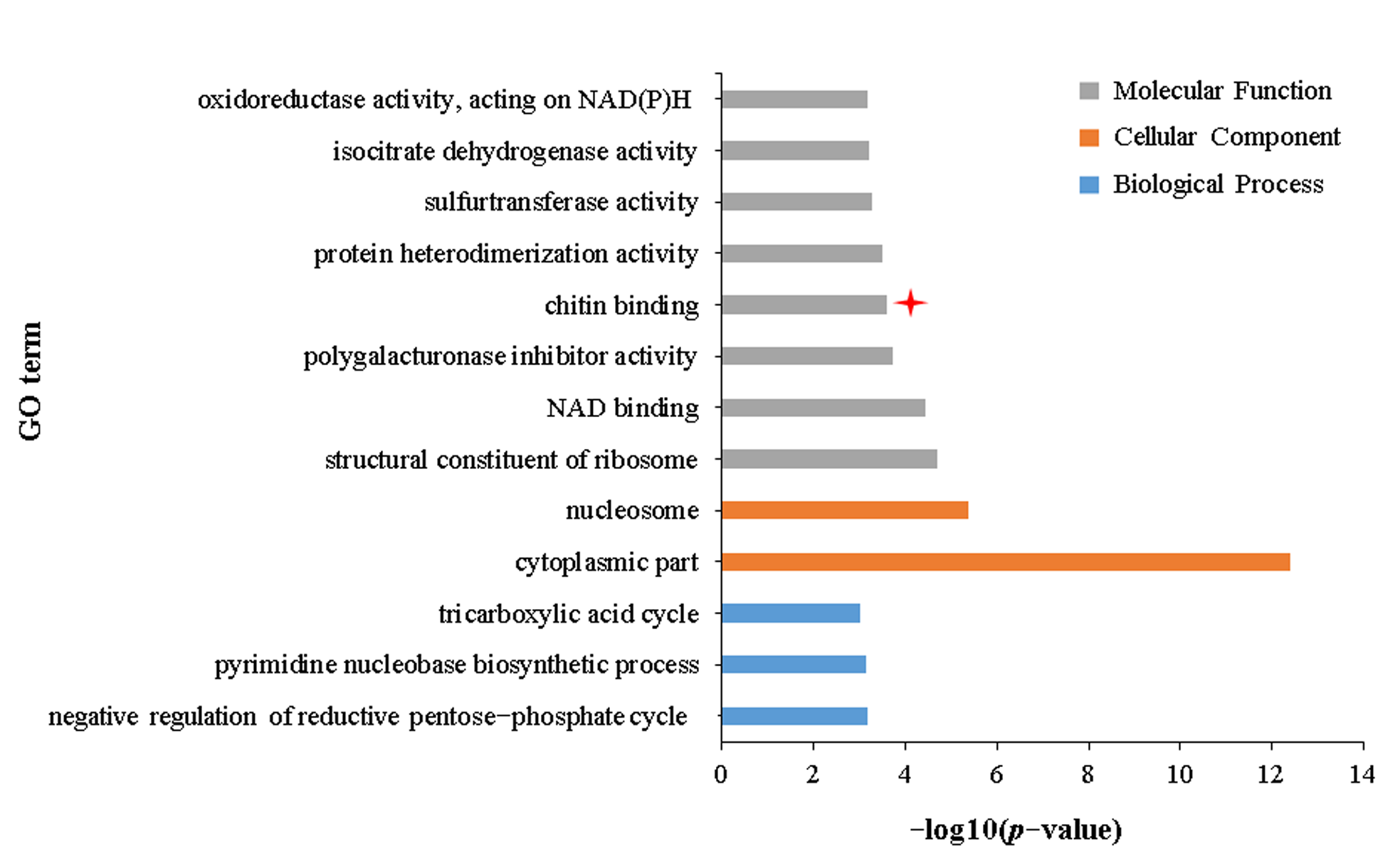

Supplement: Supplementary file 1 [file plants-12-02855-s001.zip › plants-2467175-supplementary/Supplementary materials/Supplementary Figures/Figure S4.tif]

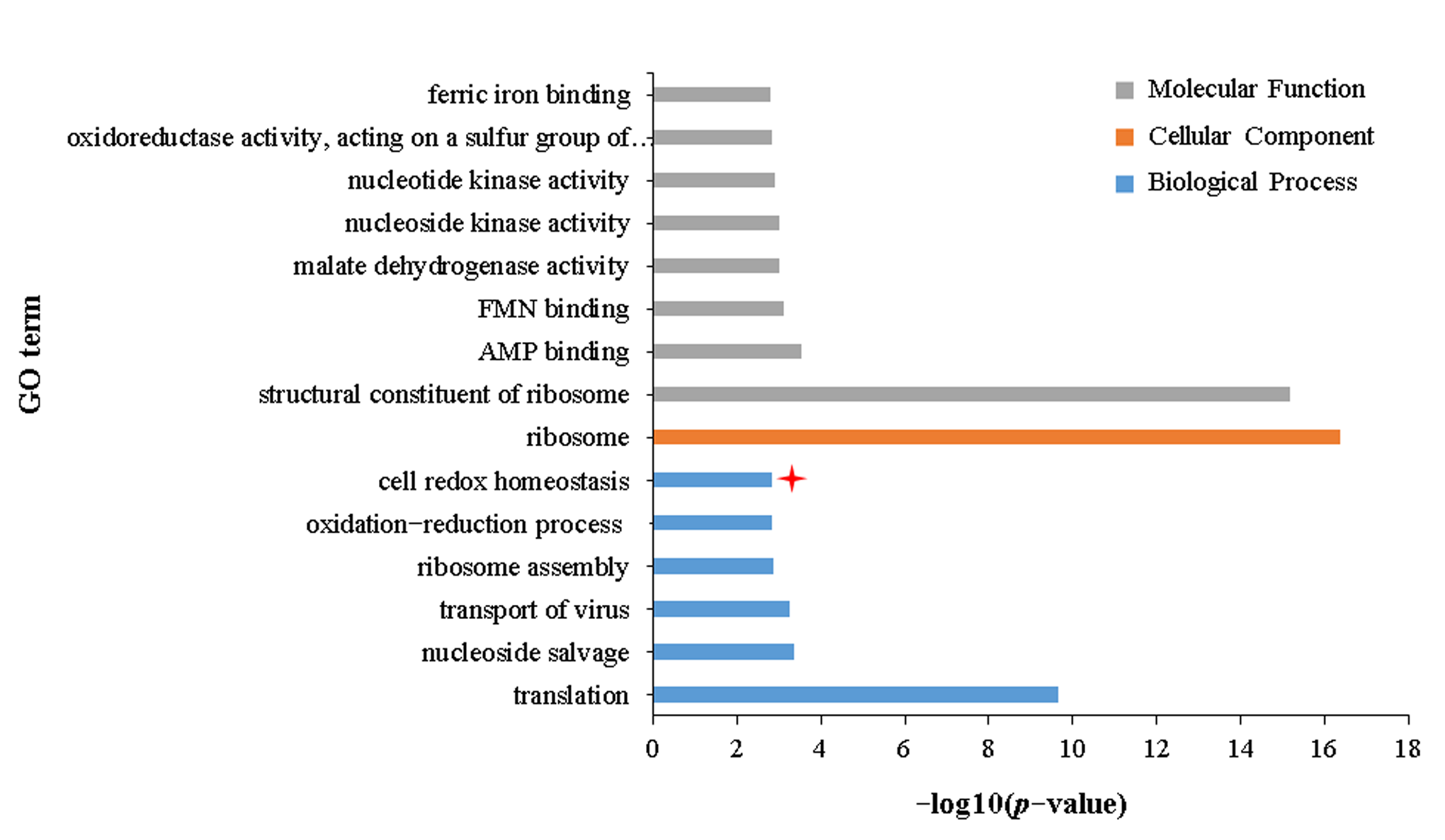

Supplement: Supplementary file 1 [file plants-12-02855-s001.zip › plants-2467175-supplementary/Supplementary materials/Supplementary Figures/Figure S5.tif]

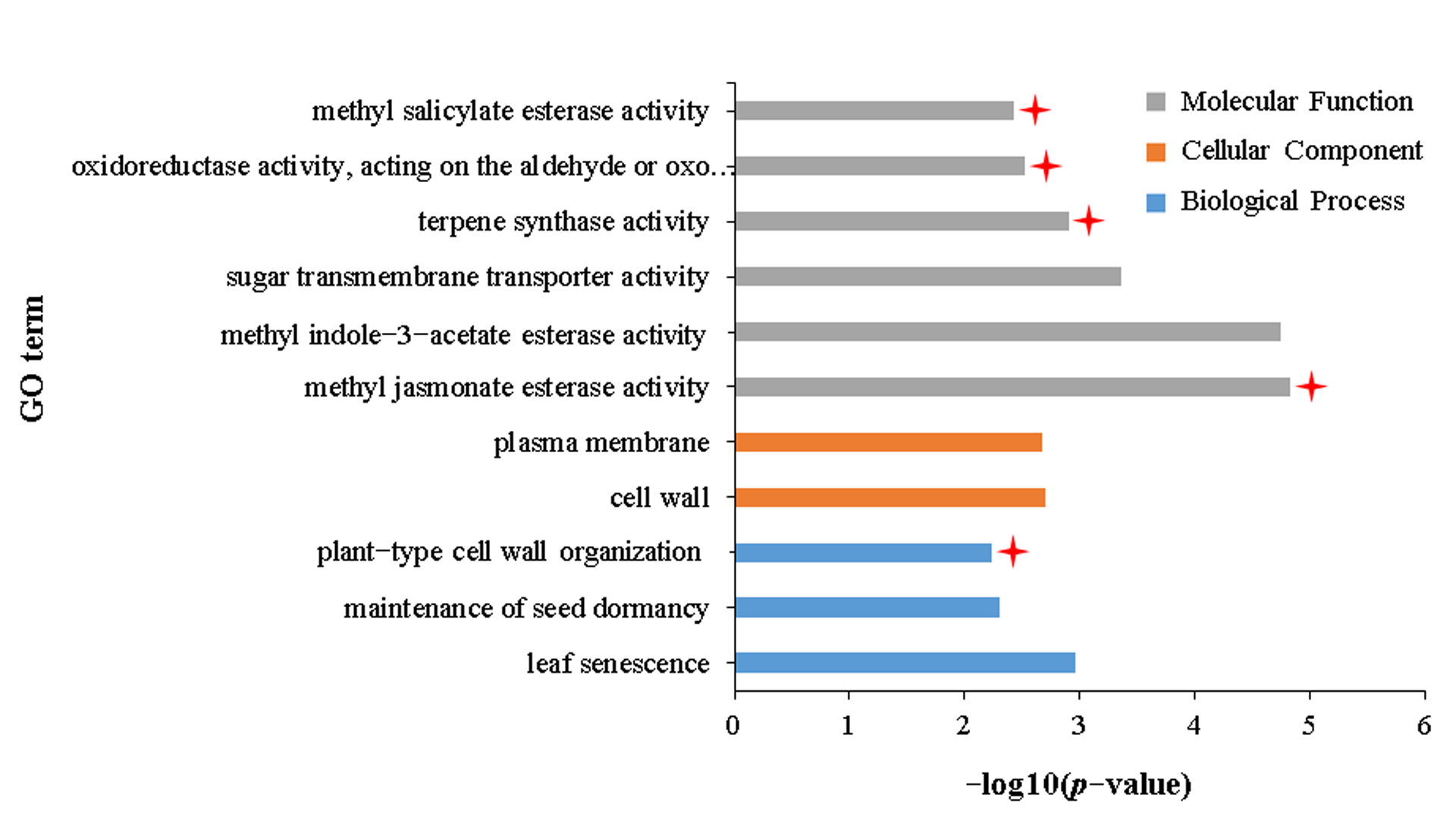

Supplement: Supplementary file 1 [file plants-12-02855-s001.zip › plants-2467175-supplementary/Supplementary materials/Supplementary Figures/Figure S8.tif]

(A)

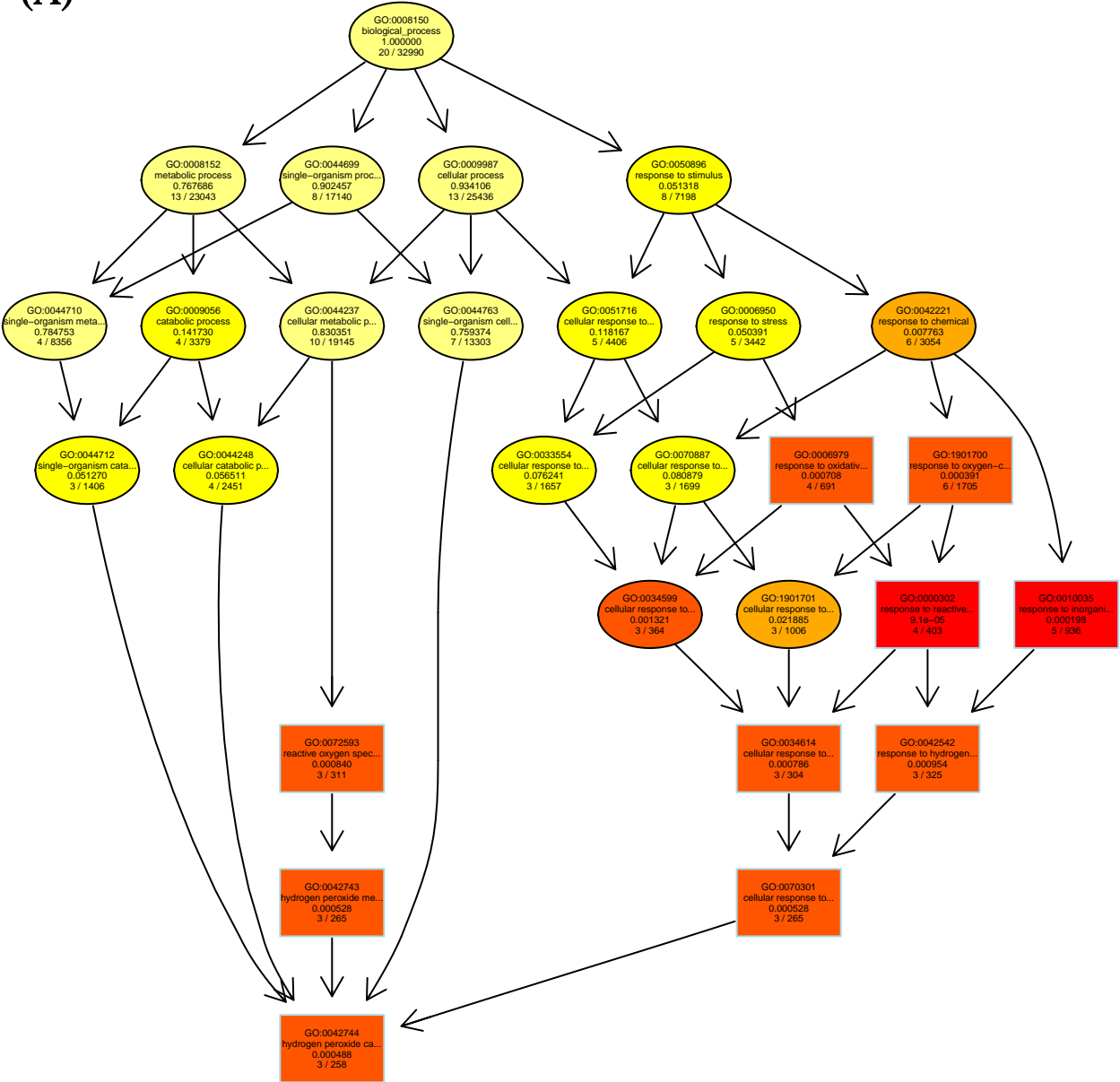

**(B)**

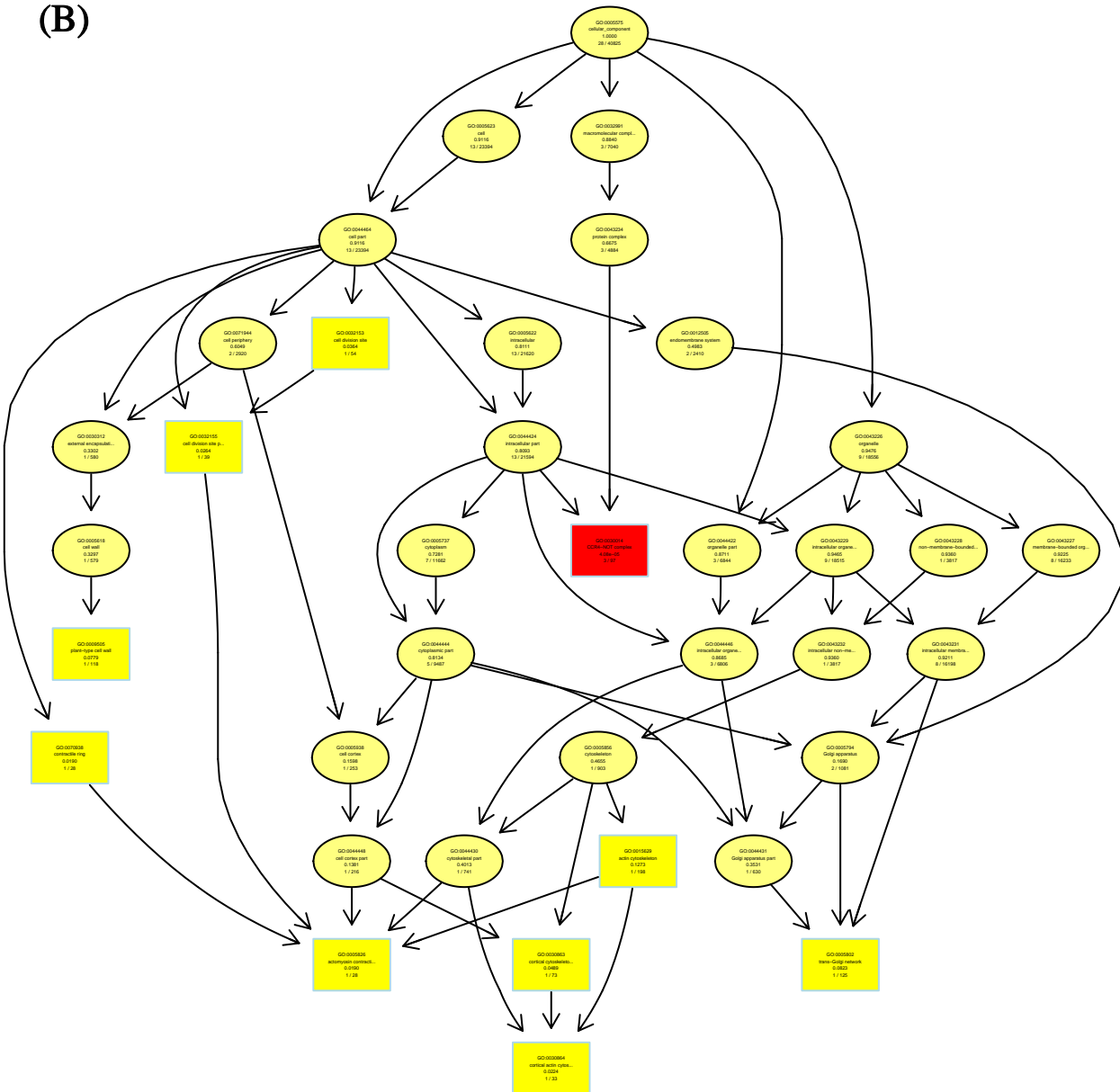

(C)

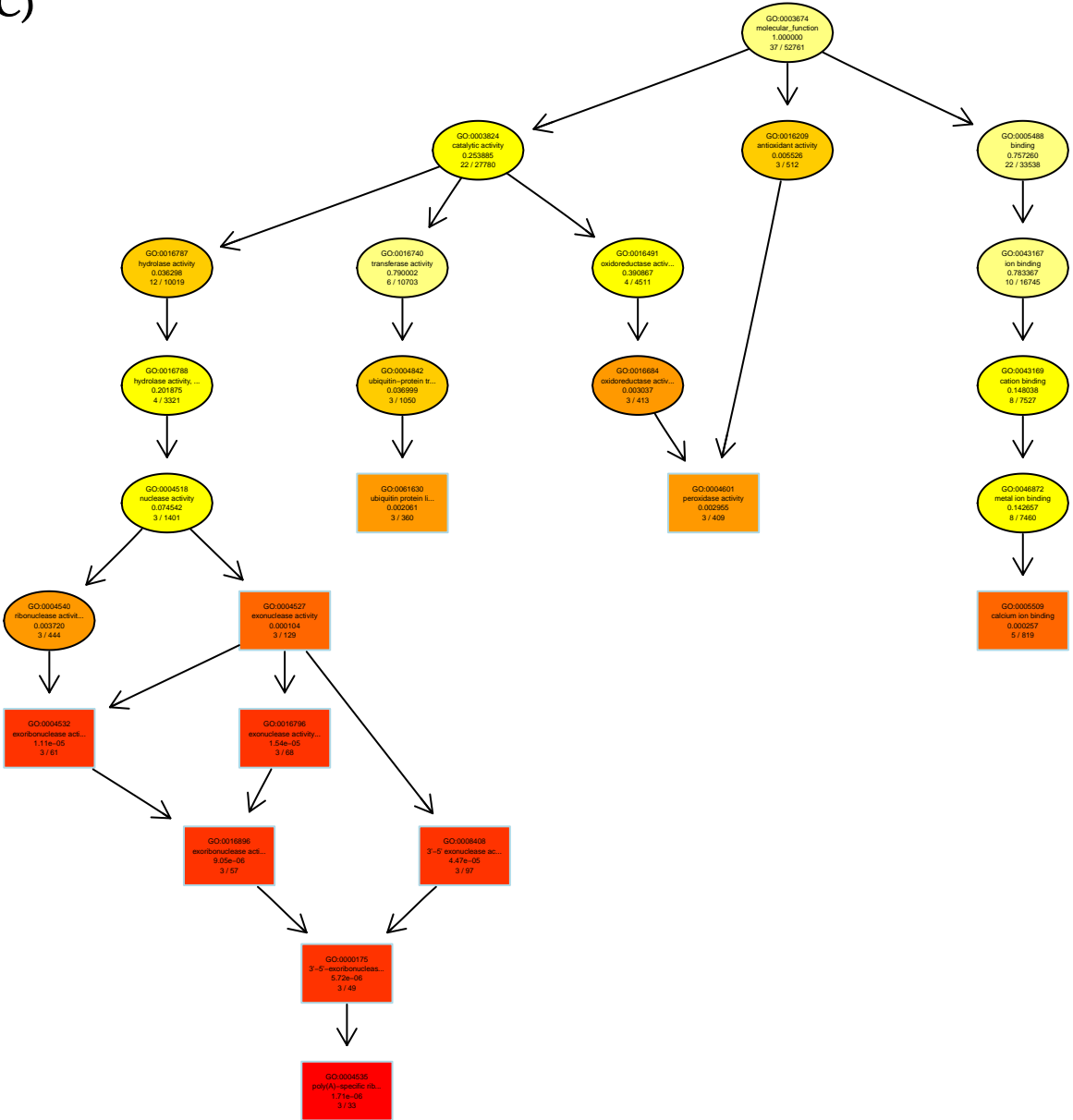

Supplement: Supplementary file 1 [file plants-12-02855-s001.zip › plants-2467175-supplementary/Supplementary materials/Supplementary Files/Supplementary File 1.pdf]

(A)

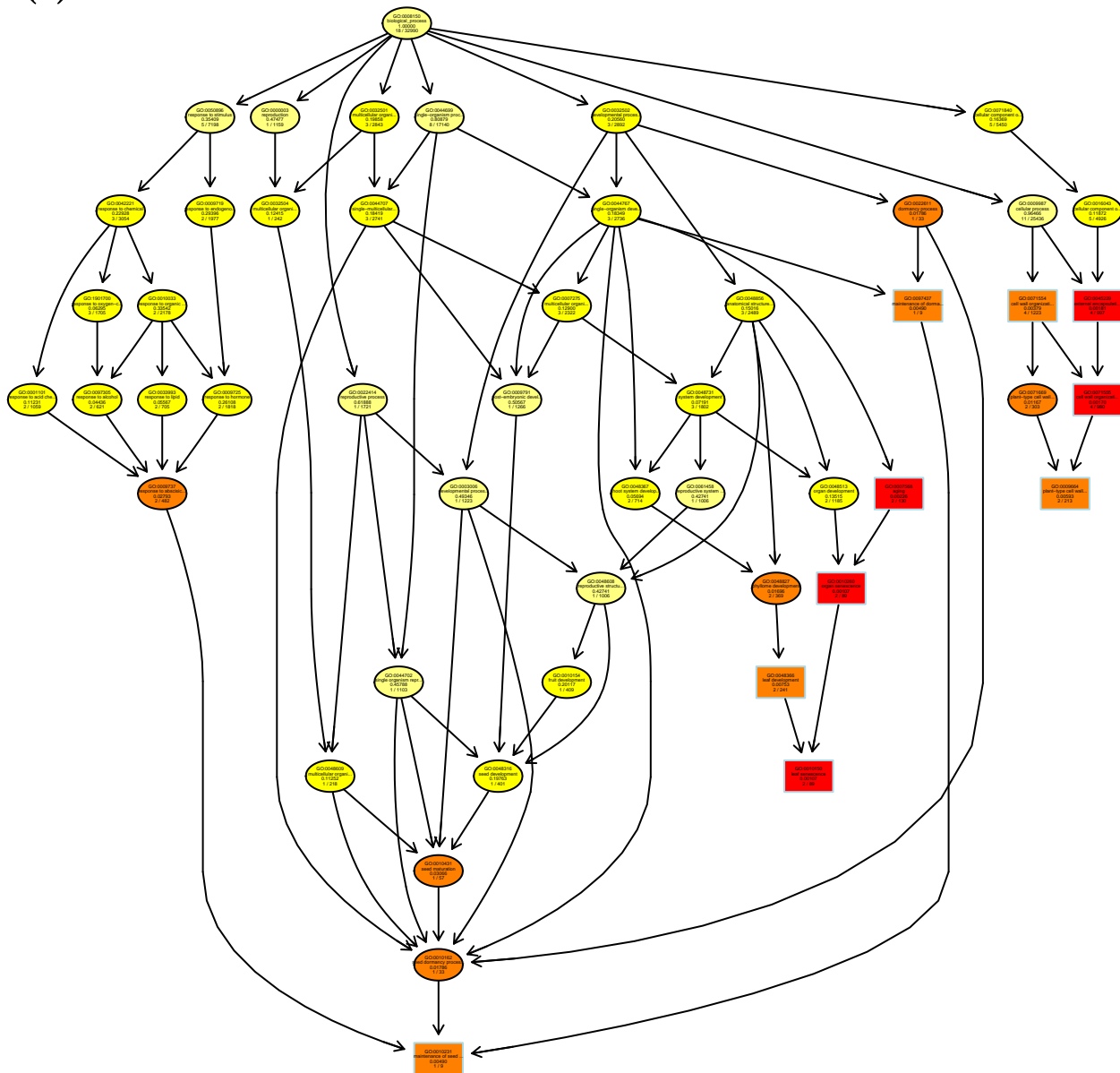

(B)

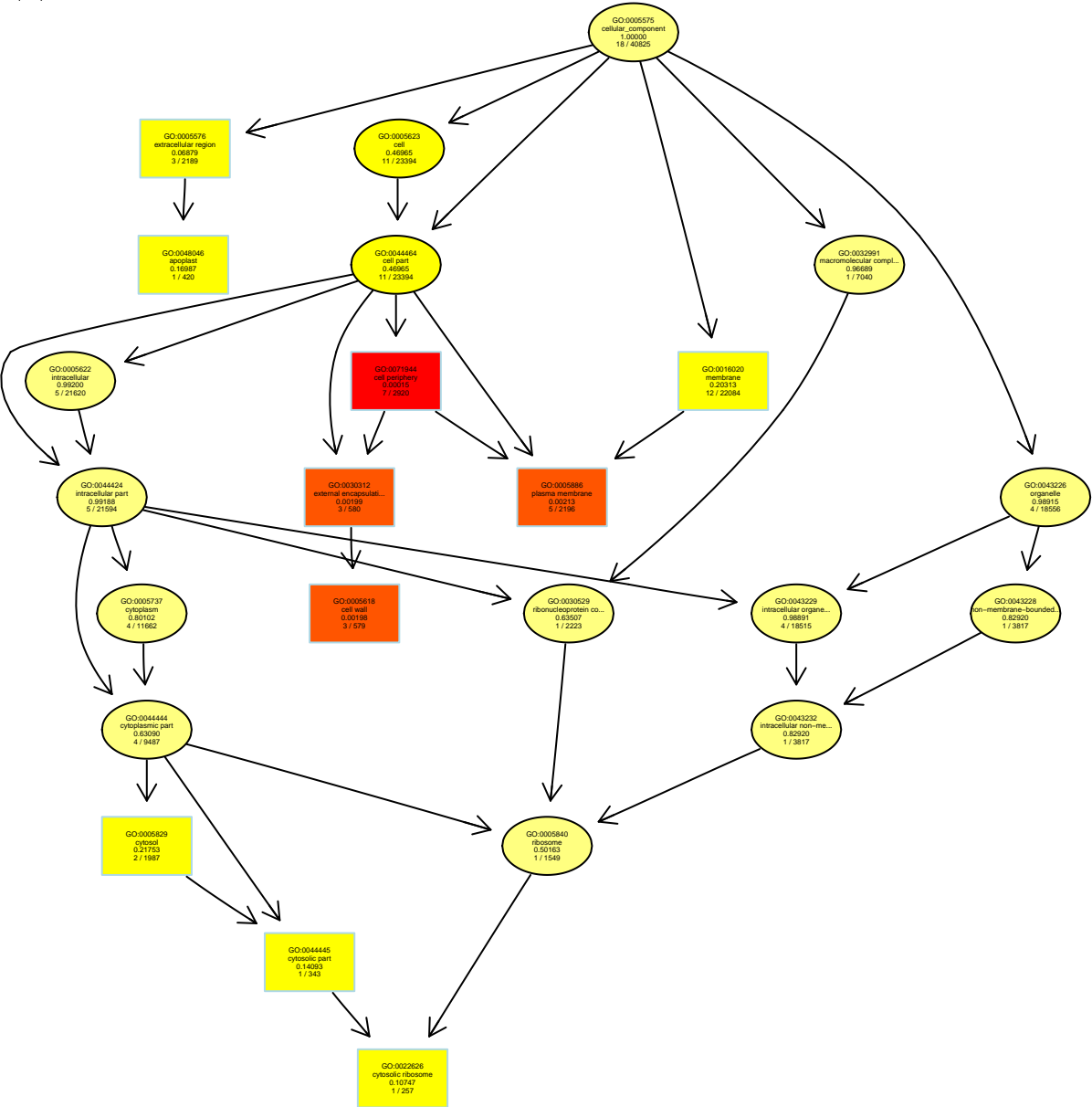

(C)

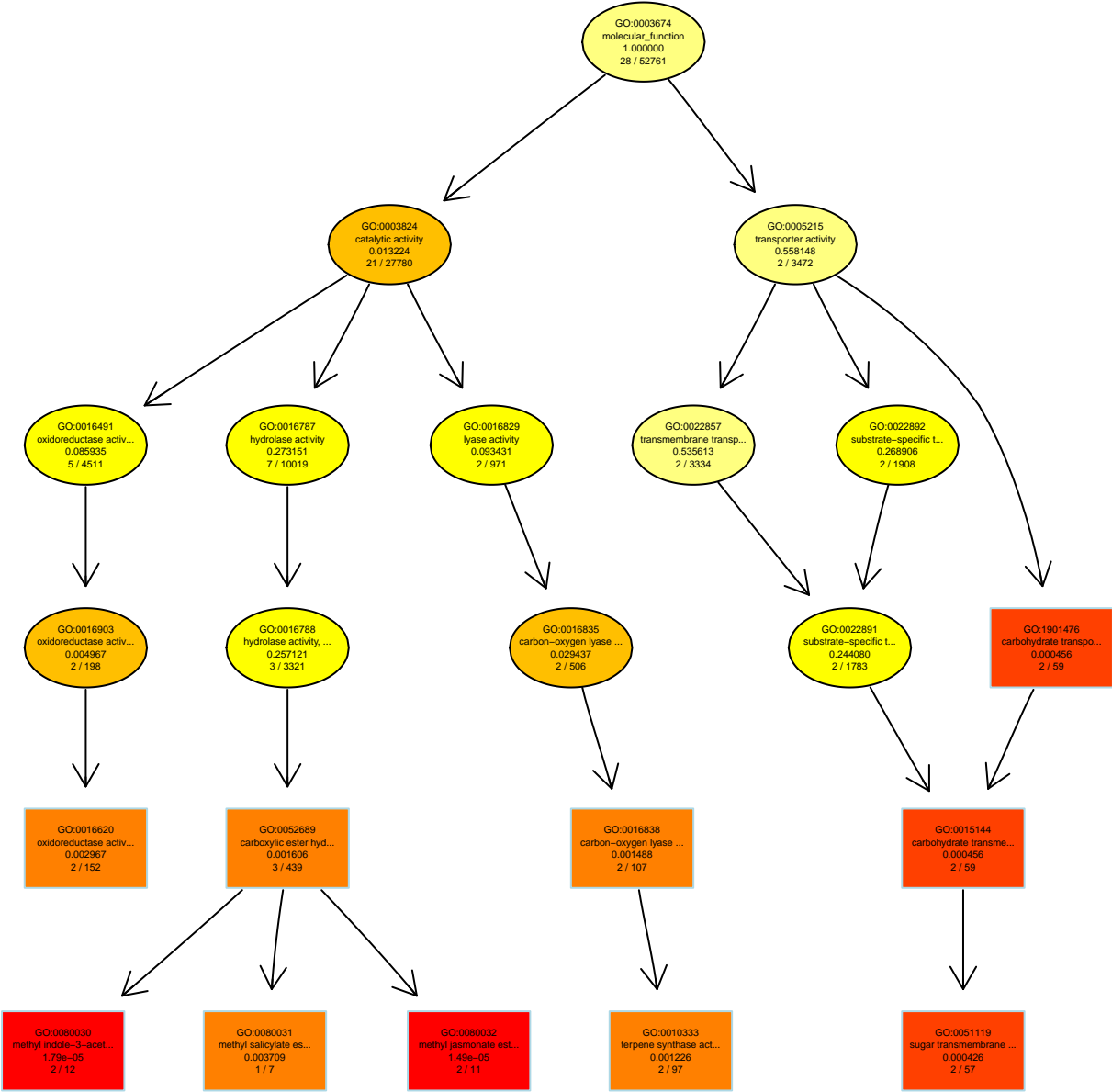

Supplement: Supplementary file 1 [file plants-12-02855-s001.zip › plants-2467175-supplementary/Supplementary materials/Supplementary Files/Supplementary File 10.pdf]

(A)

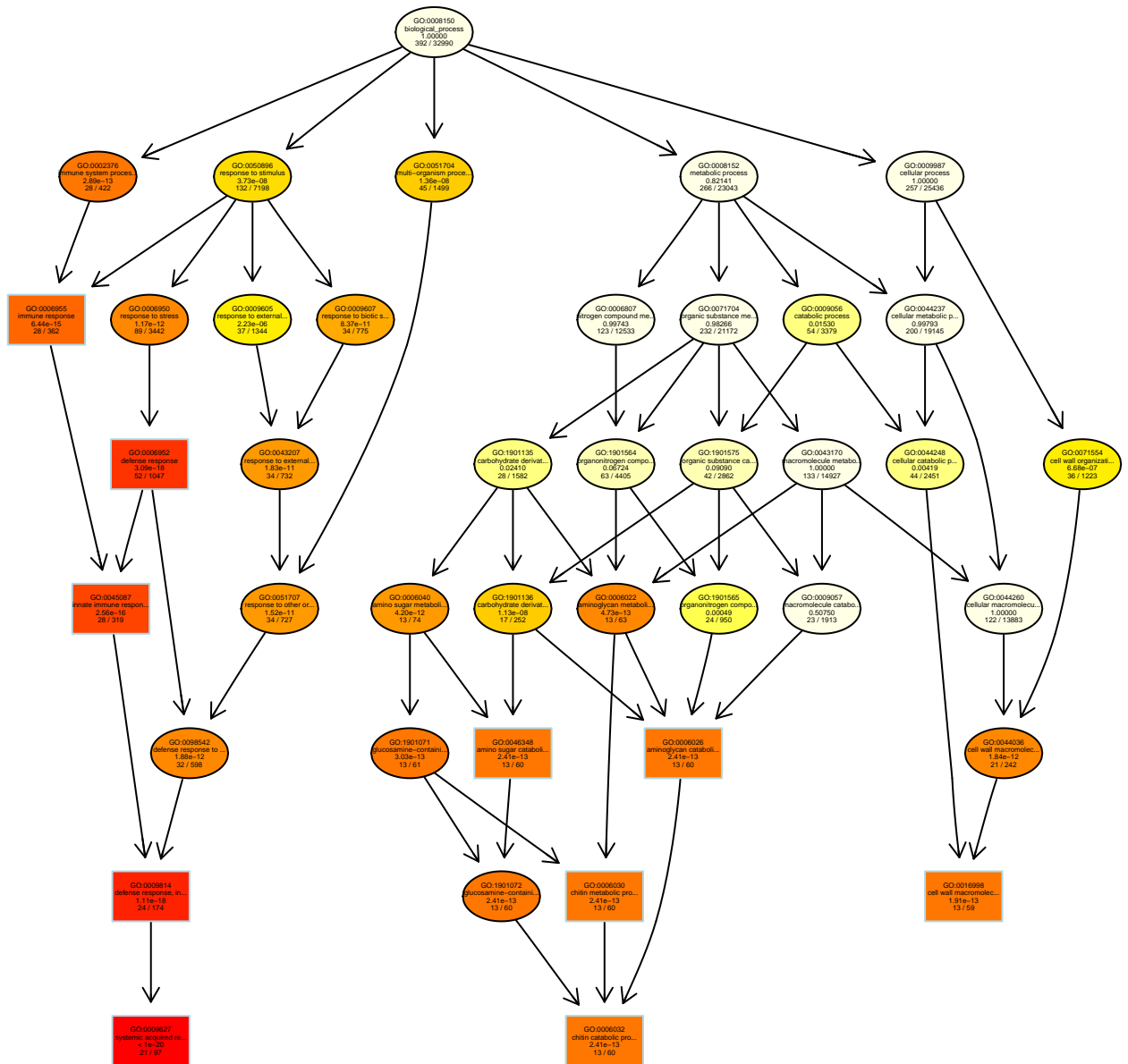

(B)

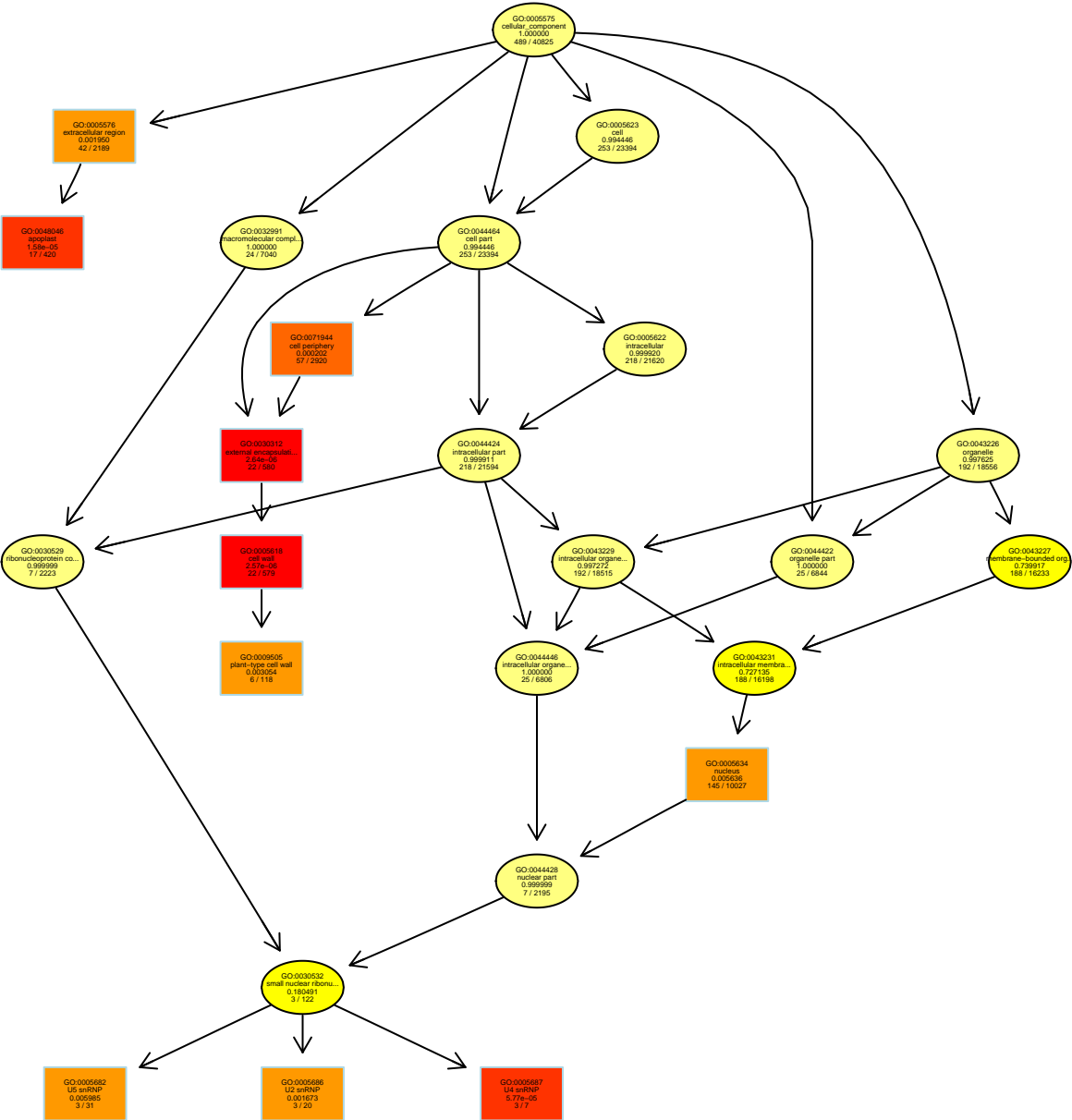

(C)

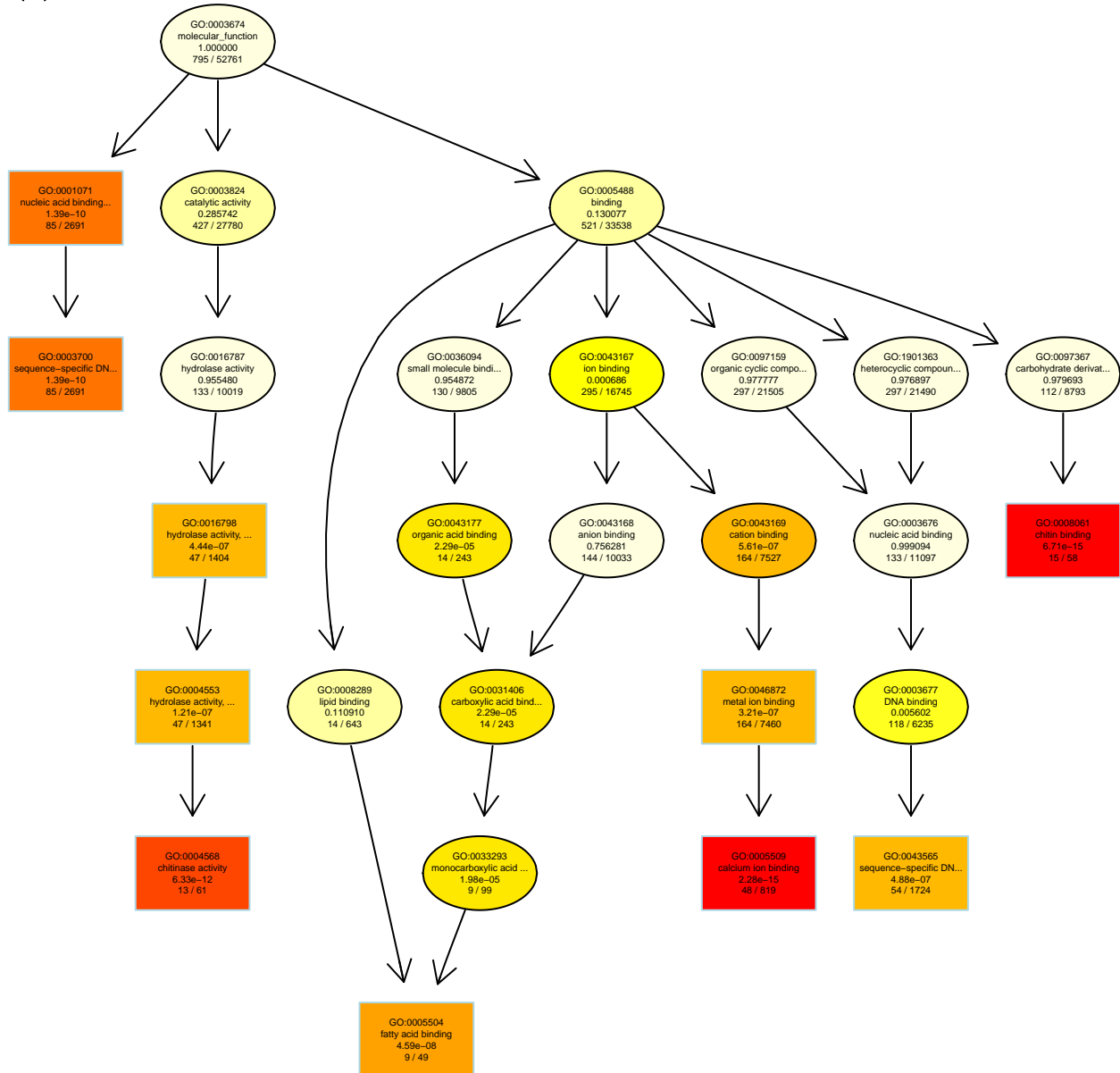

Supplement: Supplementary file 1 [file plants-12-02855-s001.zip › plants-2467175-supplementary/Supplementary materials/Supplementary Files/Supplementary File 2.pdf]

(A)

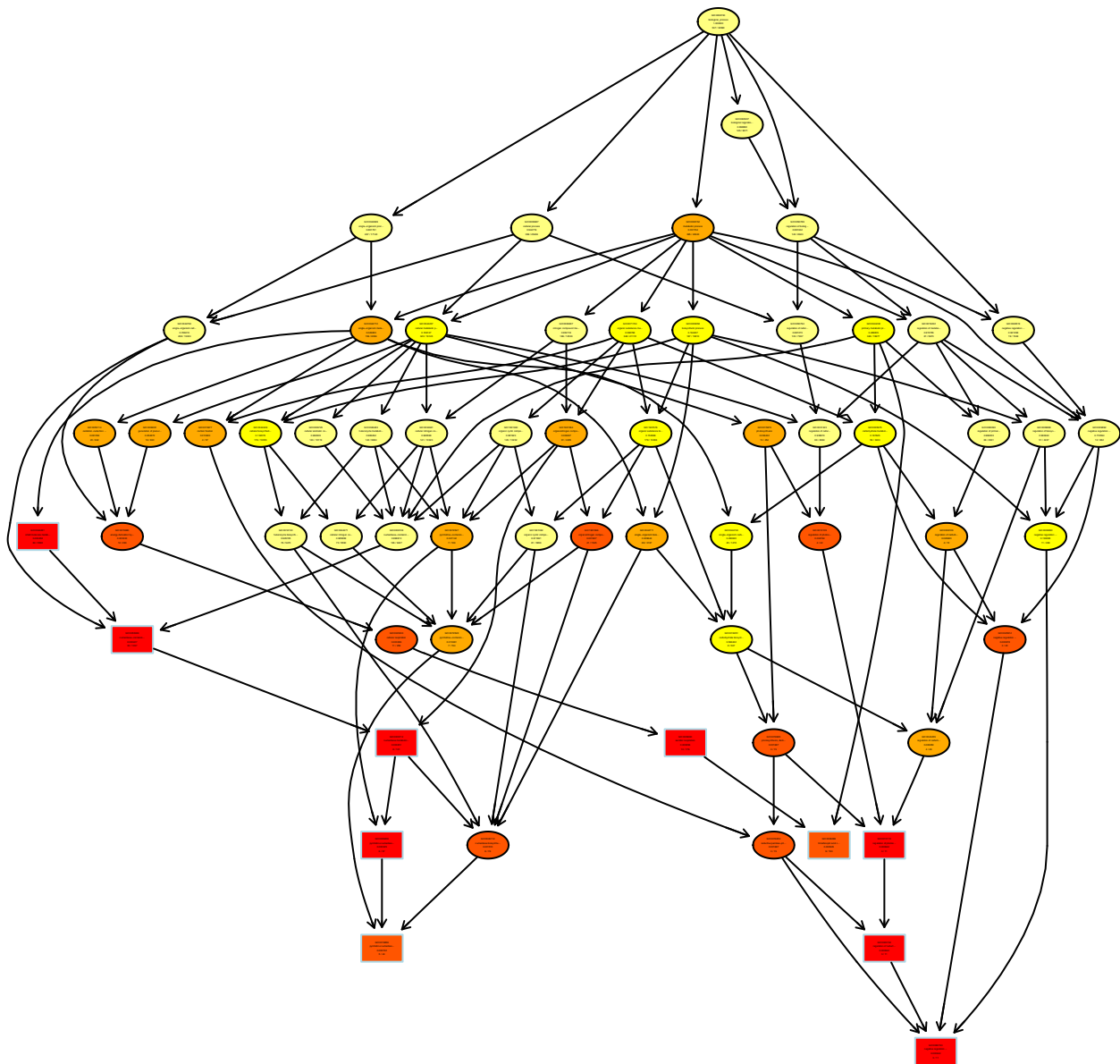

**(B)**

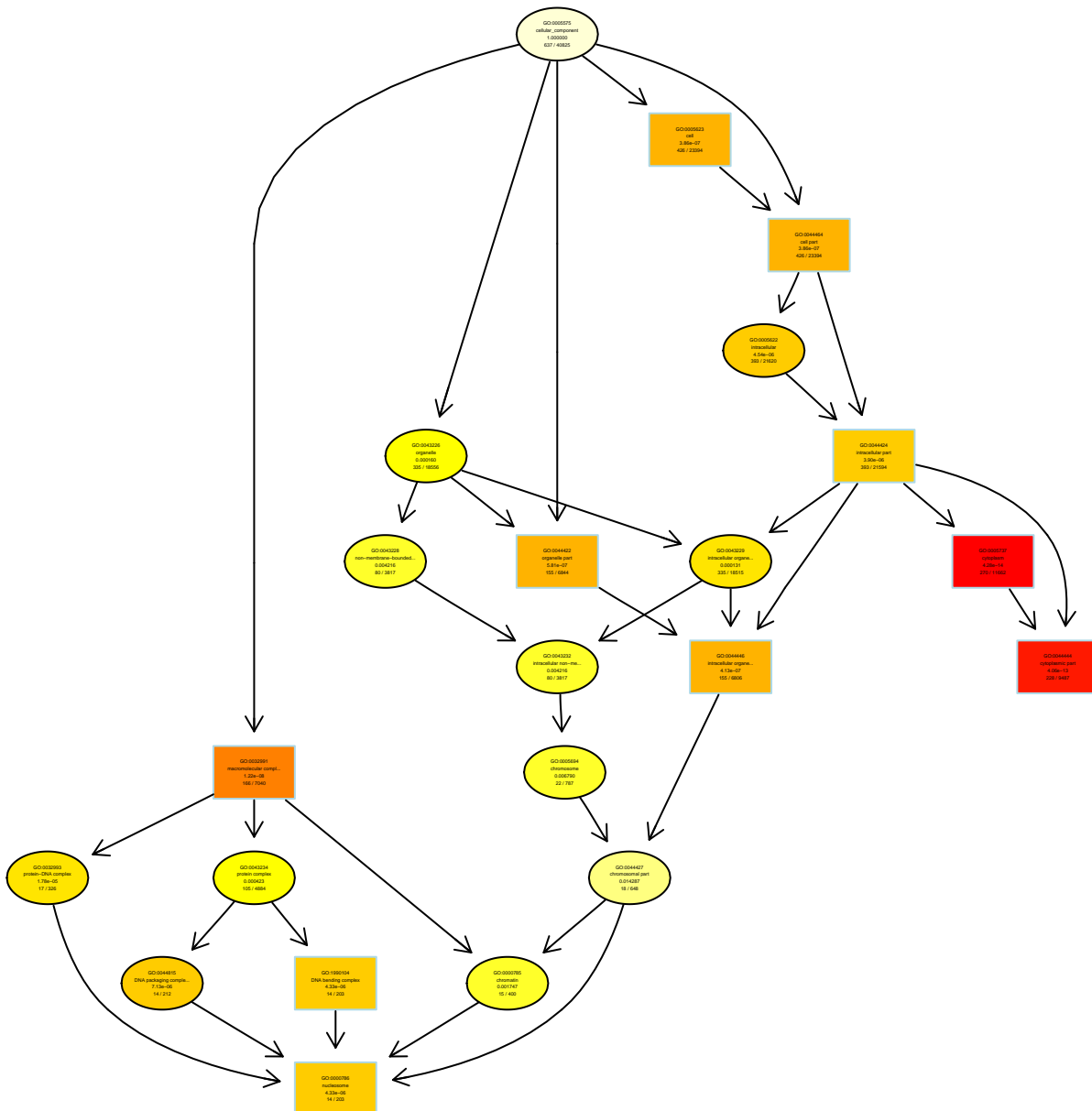

(C)

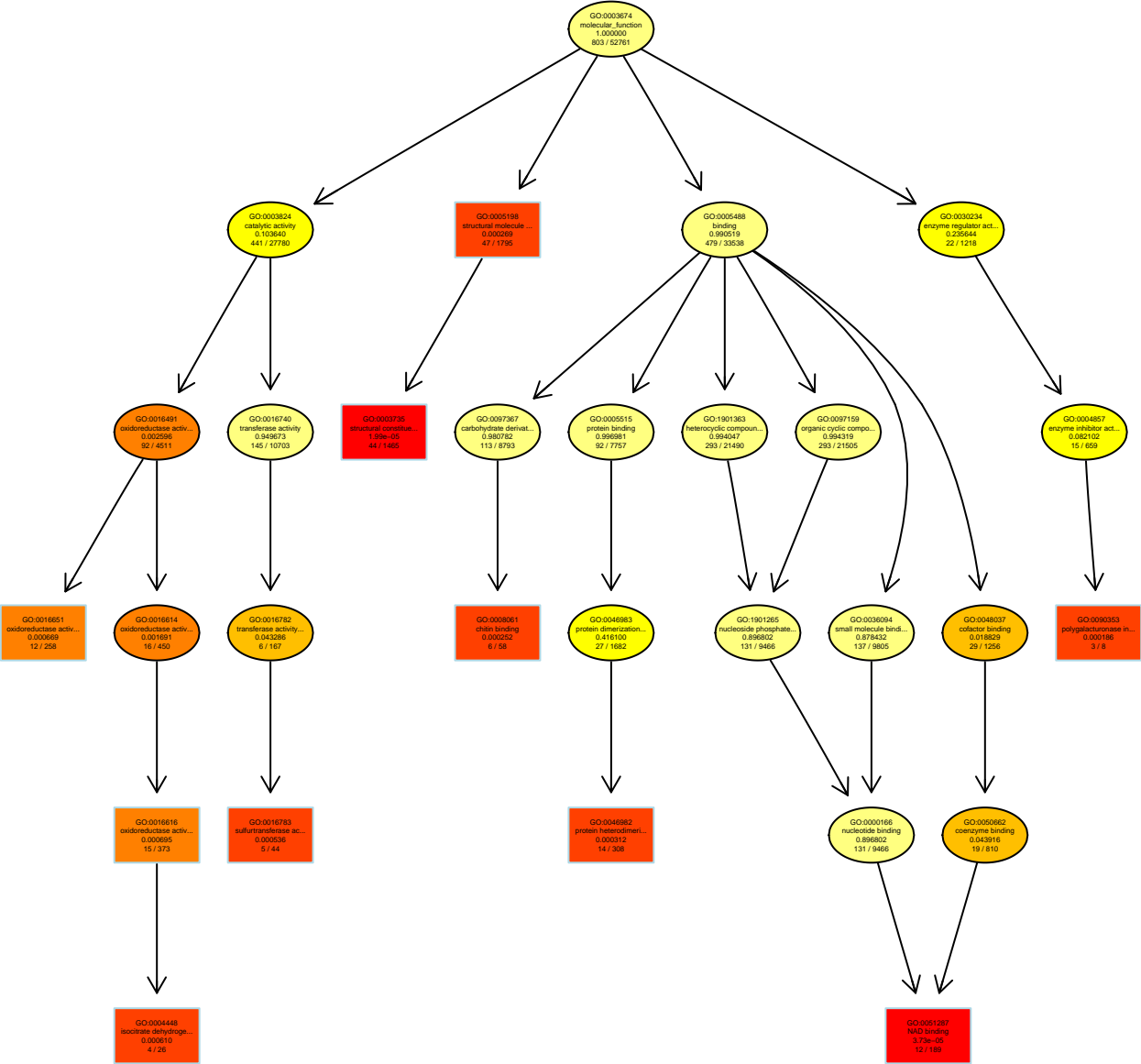

Supplement: Supplementary file 1 [file plants-12-02855-s001.zip › plants-2467175-supplementary/Supplementary materials/Supplementary Files/Supplementary File 3.pdf]

(A)

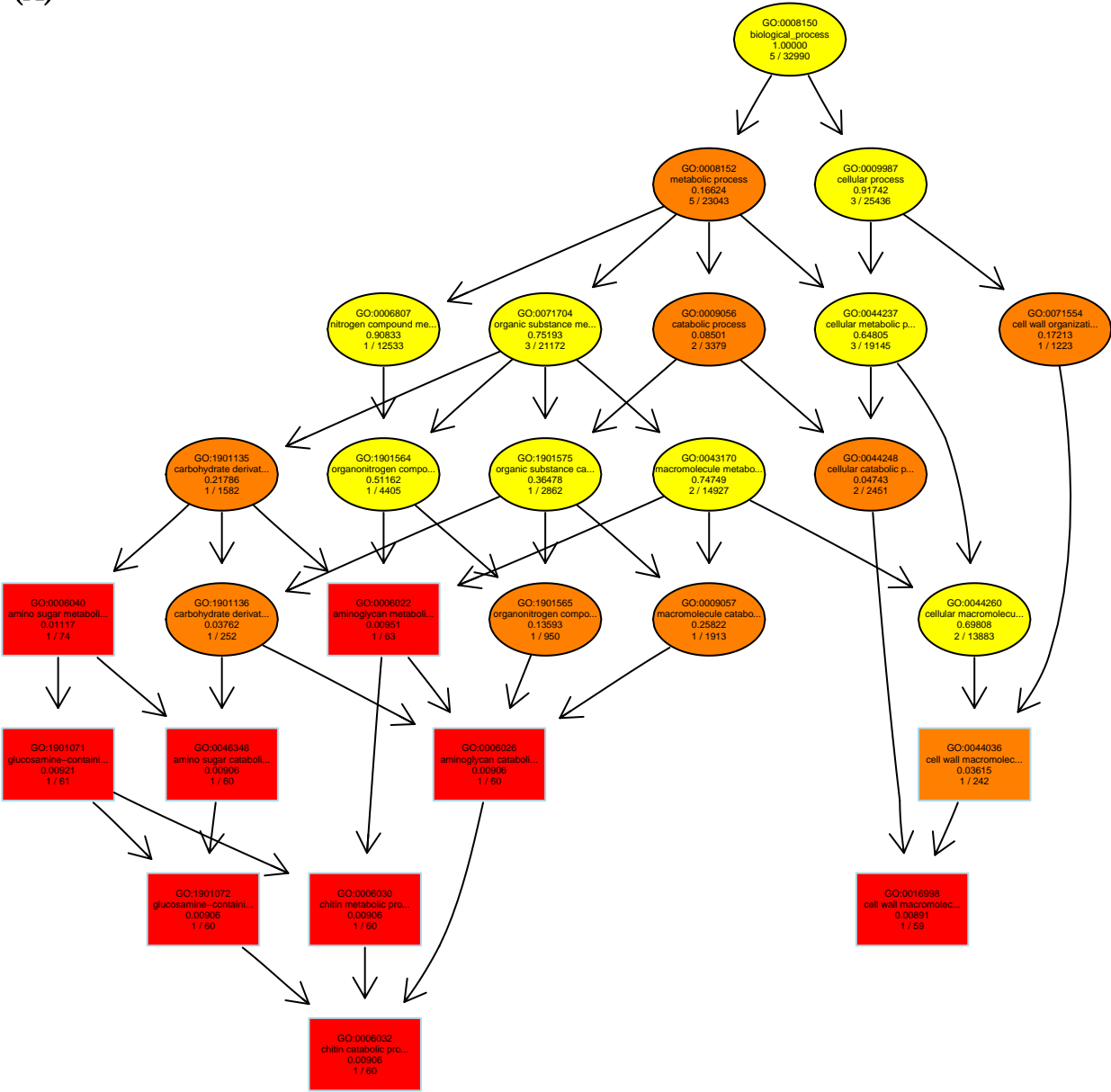

(B)

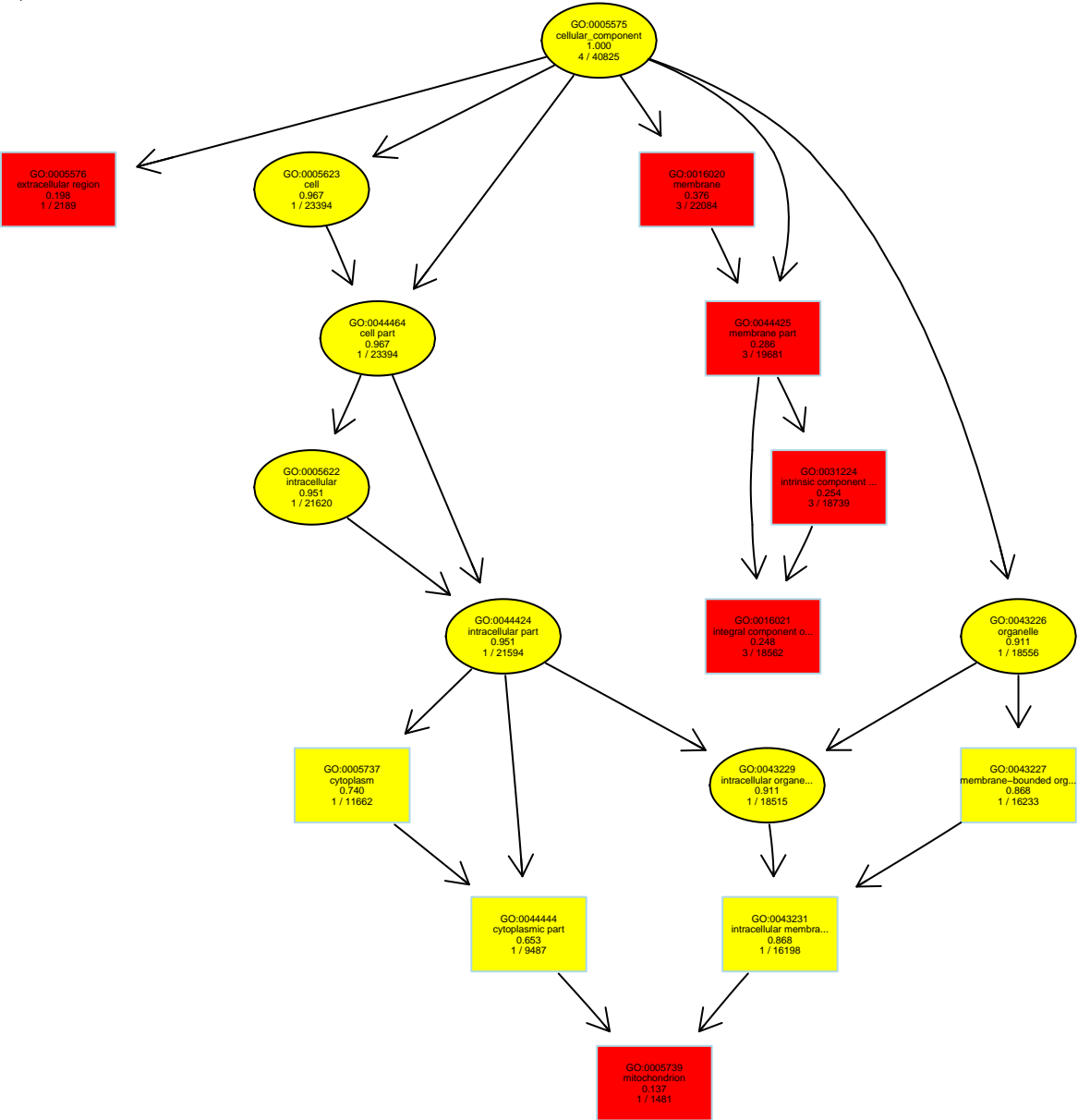

(C)

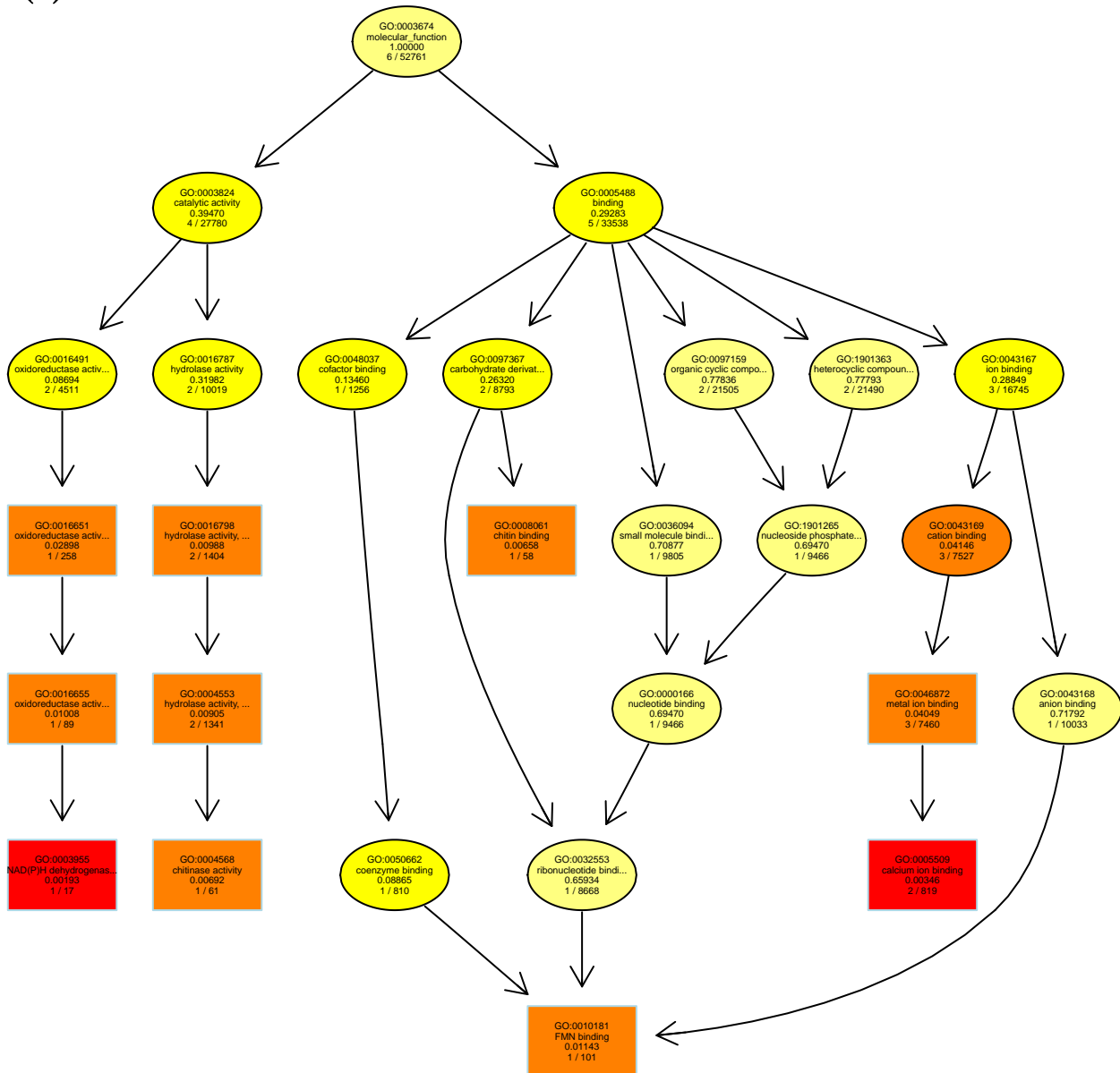

Supplement: Supplementary file 1 [file plants-12-02855-s001.zip › plants-2467175-supplementary/Supplementary materials/Supplementary Files/Supplementary File 4.pdf]

(A)

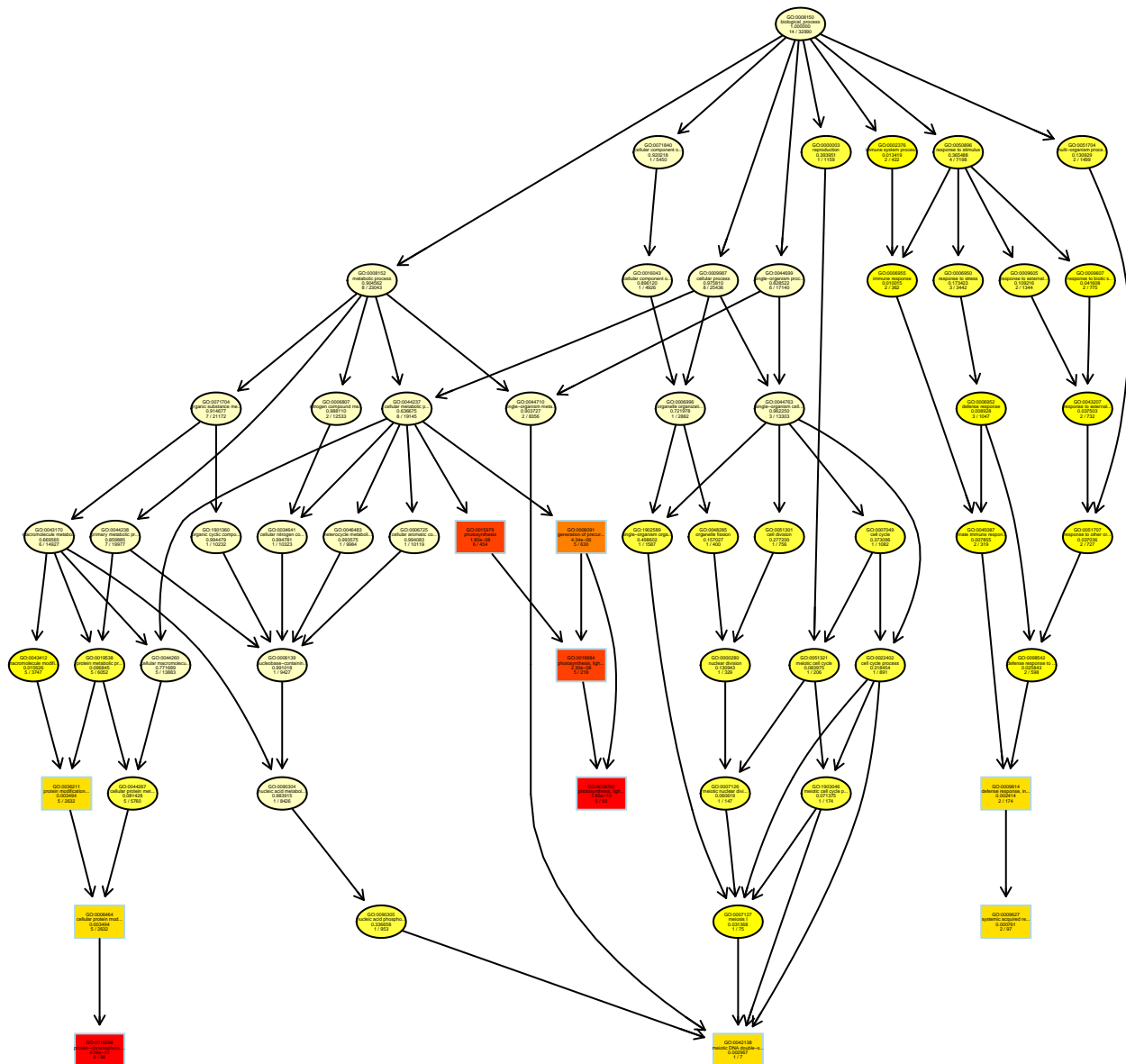

(B)

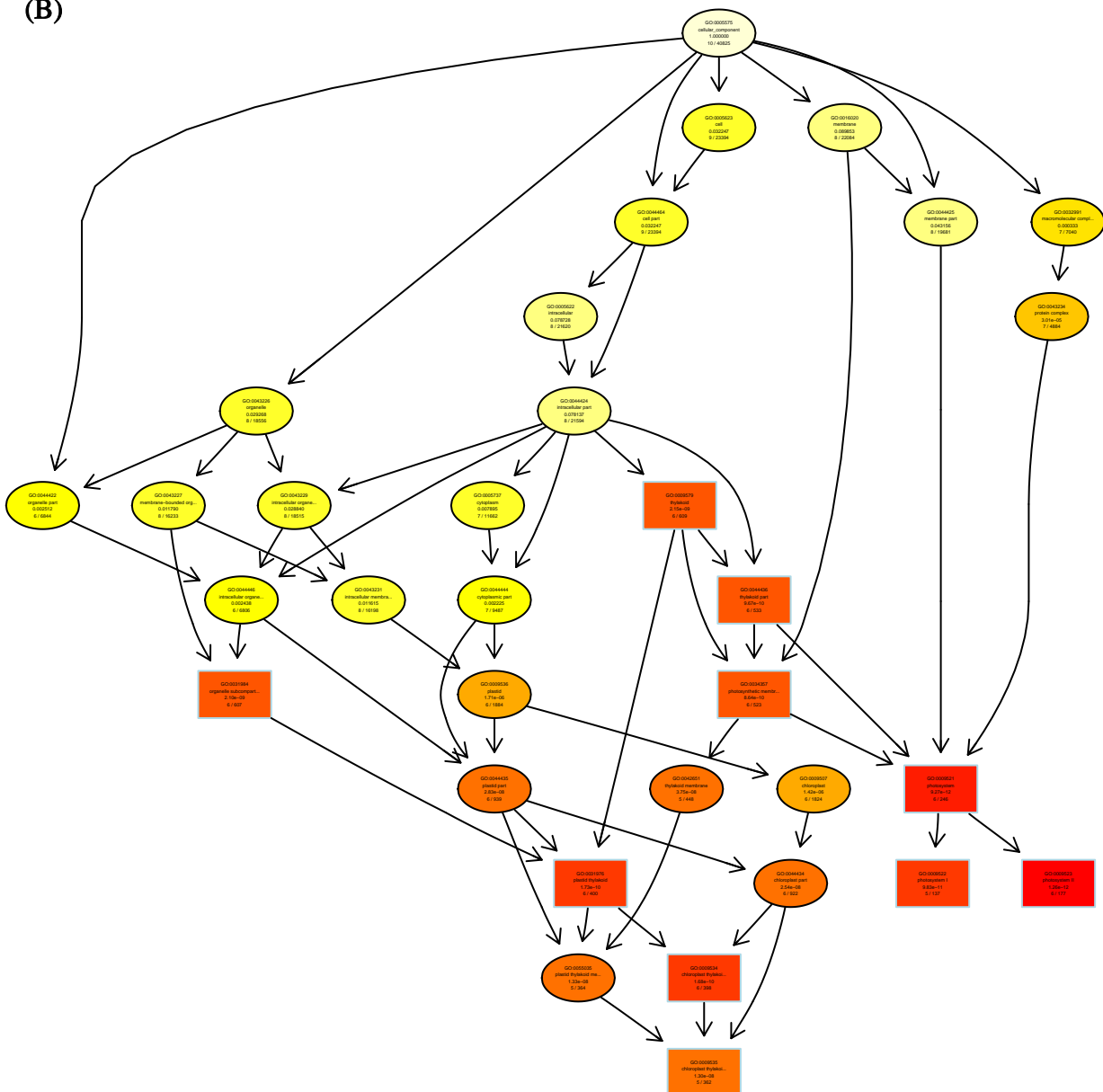

(C)

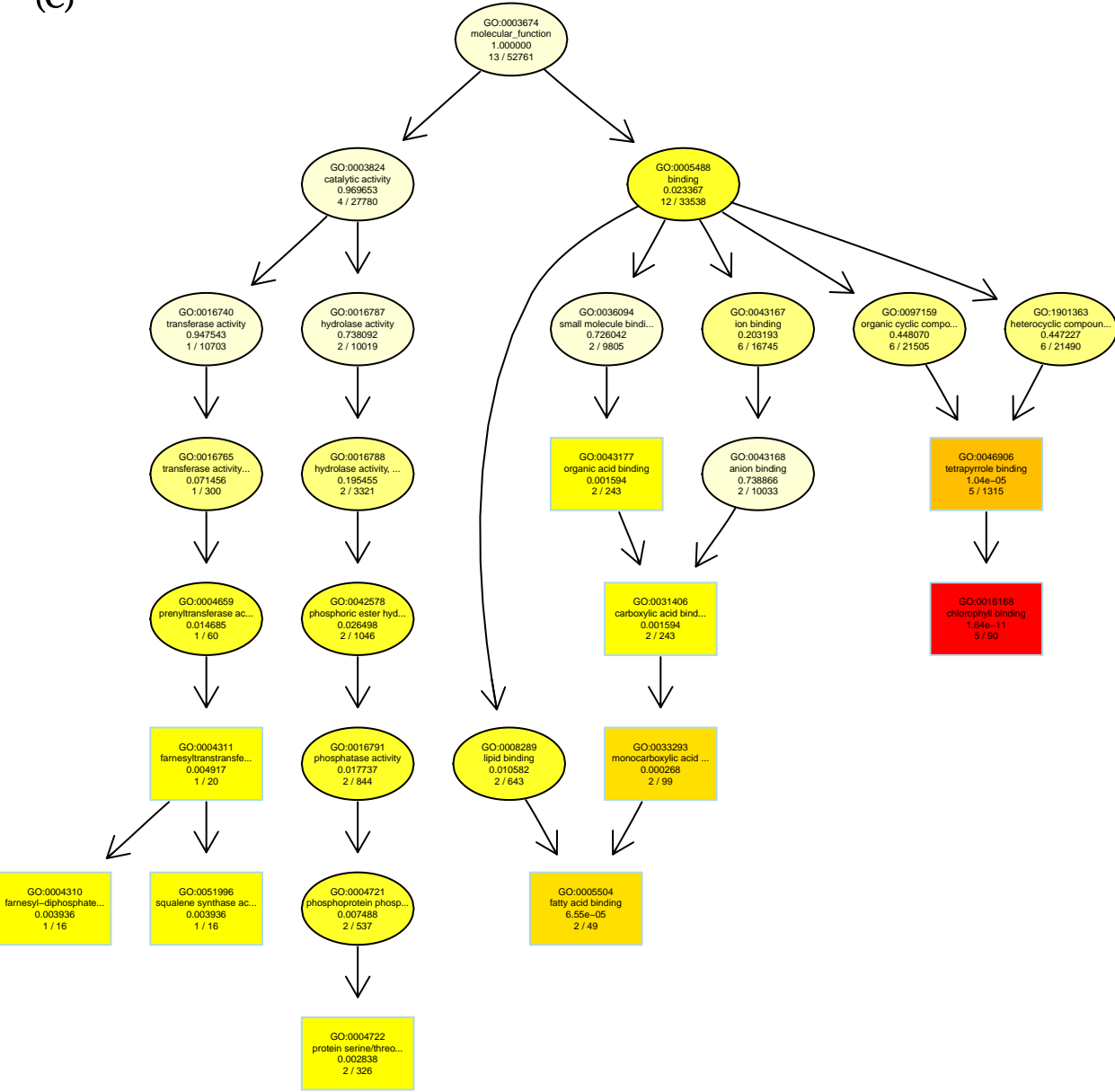

Supplement: Supplementary file 1 [file plants-12-02855-s001.zip › plants-2467175-supplementary/Supplementary materials/Supplementary Files/Supplementary File 5.pdf]

(A)

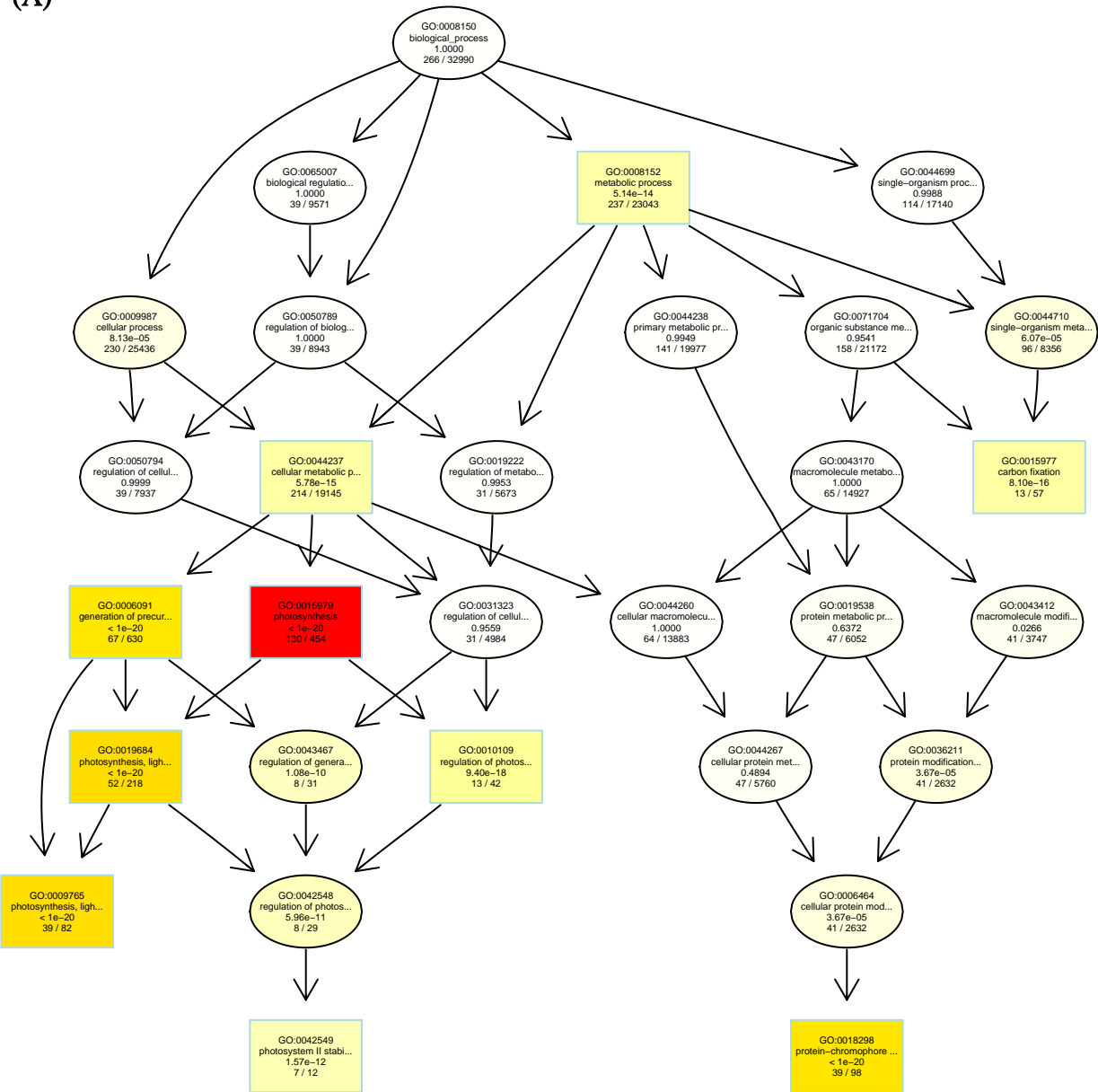

(B)

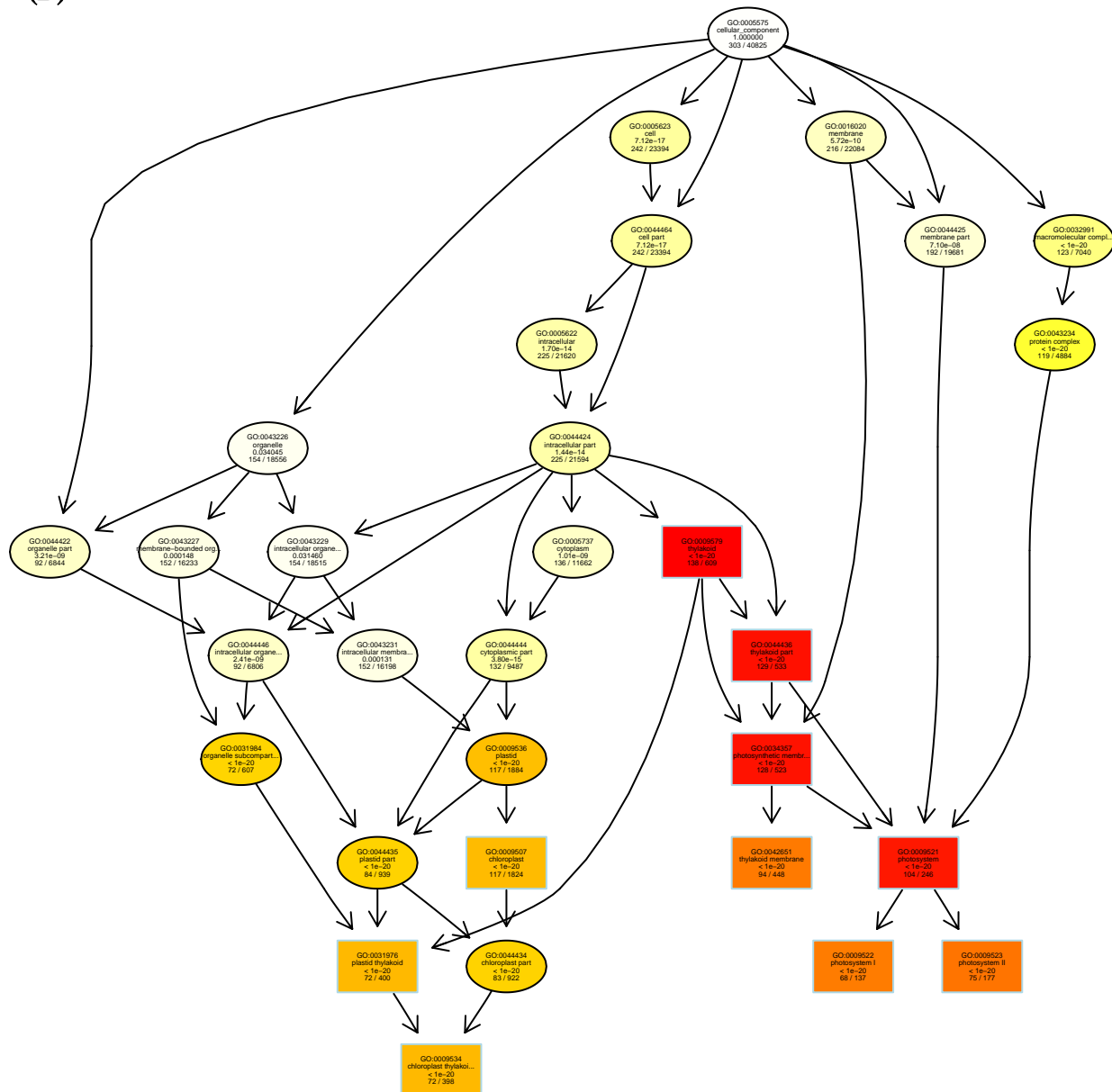

(C)

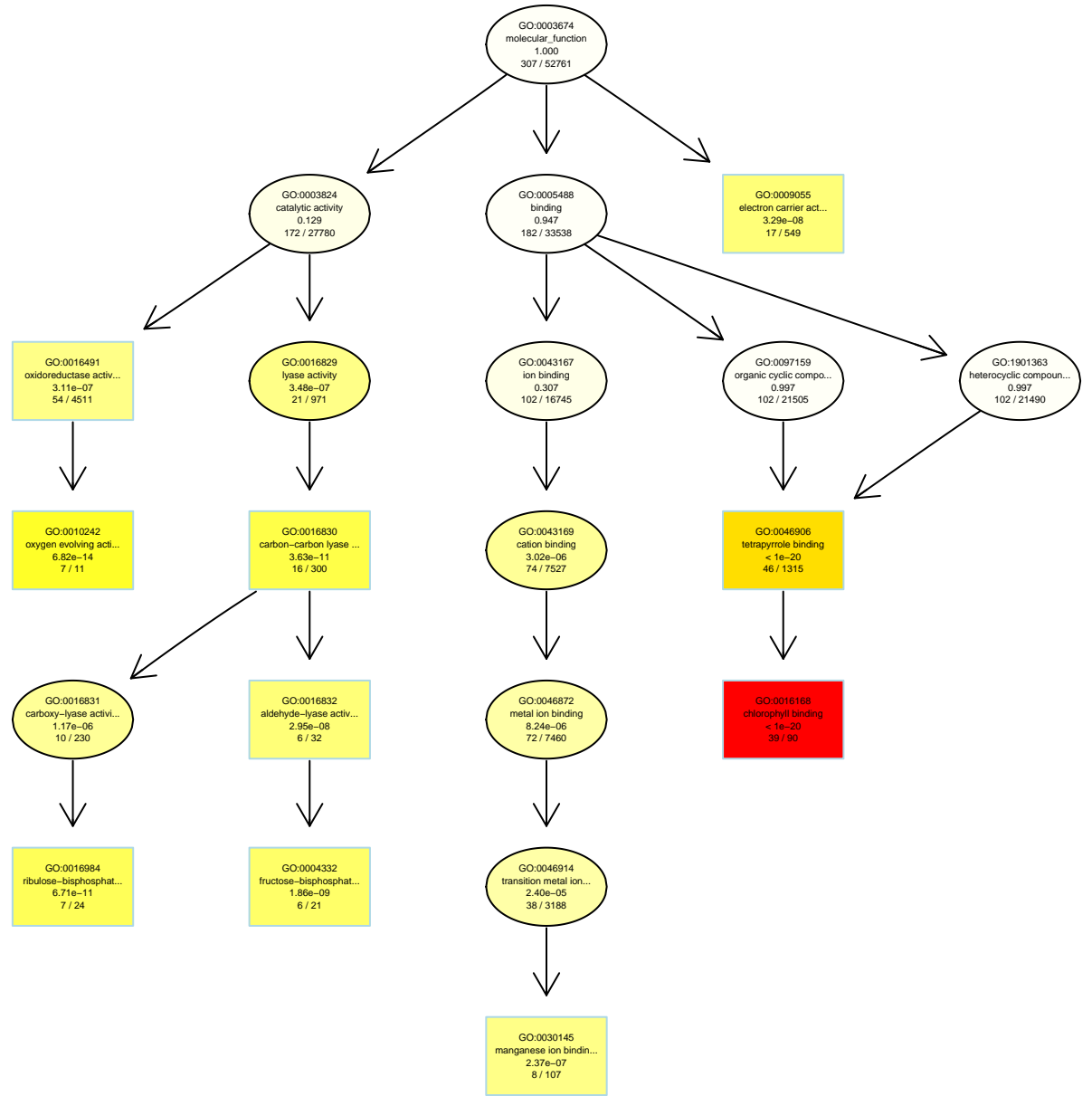

Supplement: Supplementary file 1 [file plants-12-02855-s001.zip › plants-2467175-supplementary/Supplementary materials/Supplementary Files/Supplementary File 6.pdf]

(A)

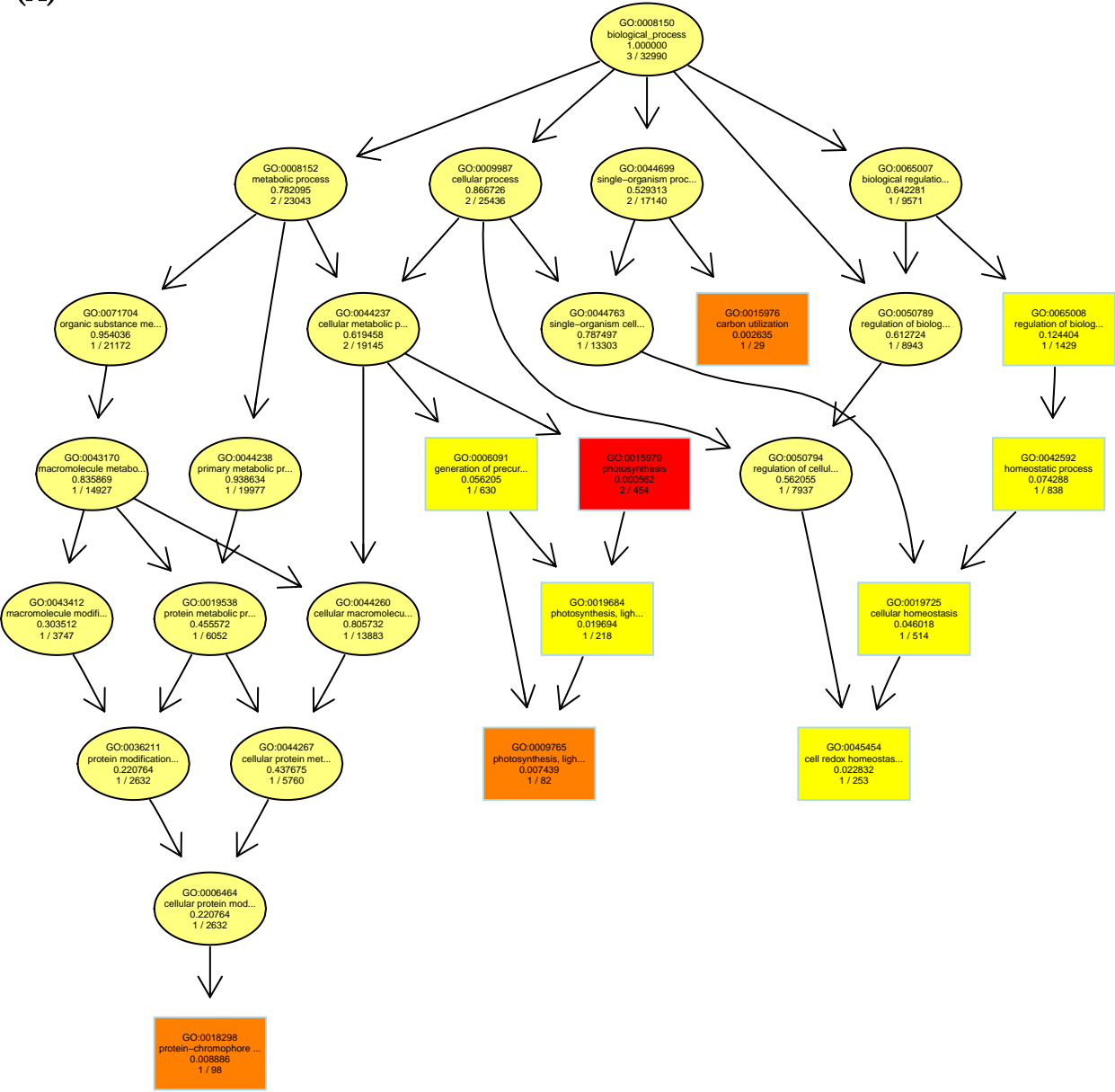

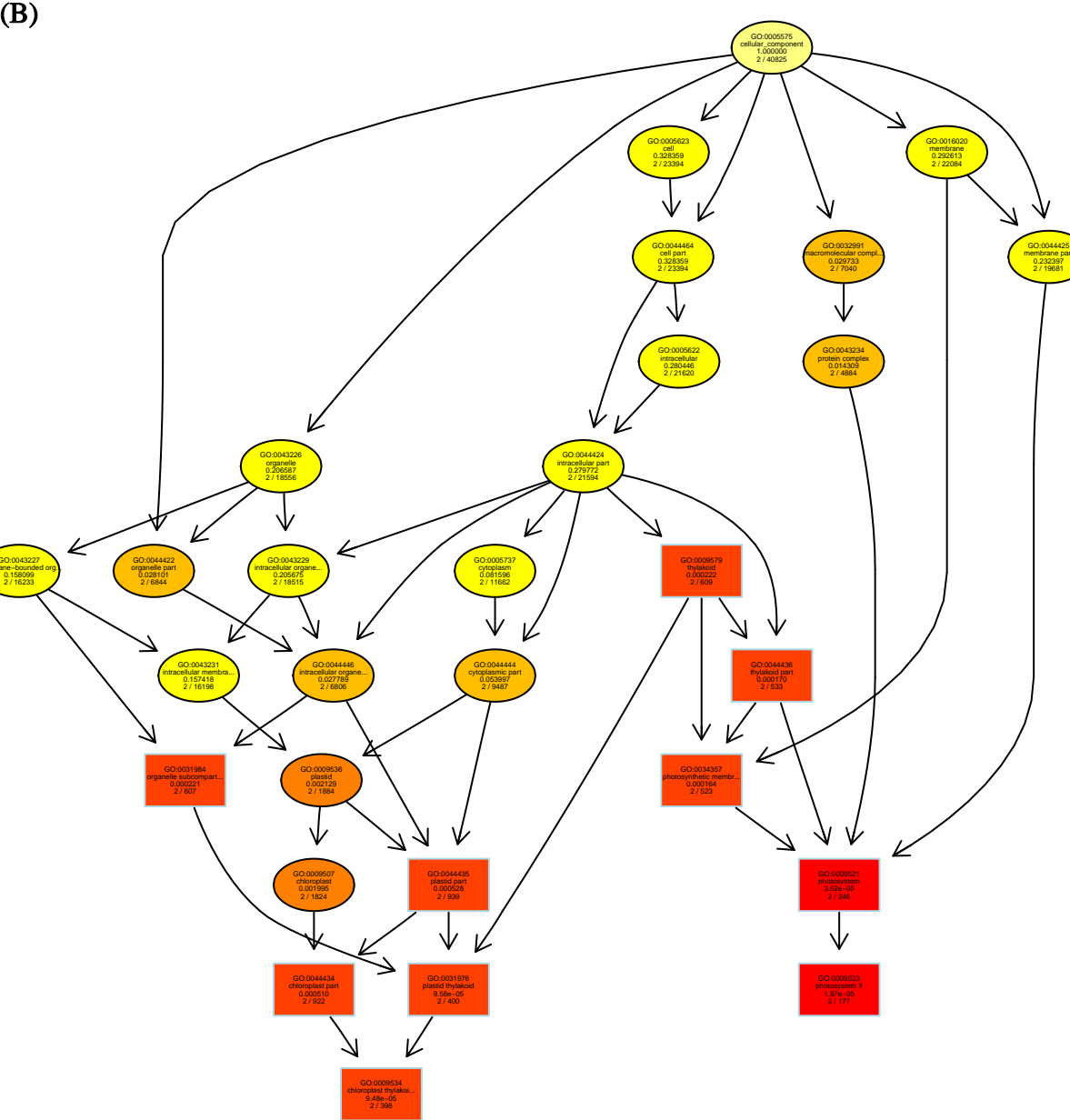

(C)

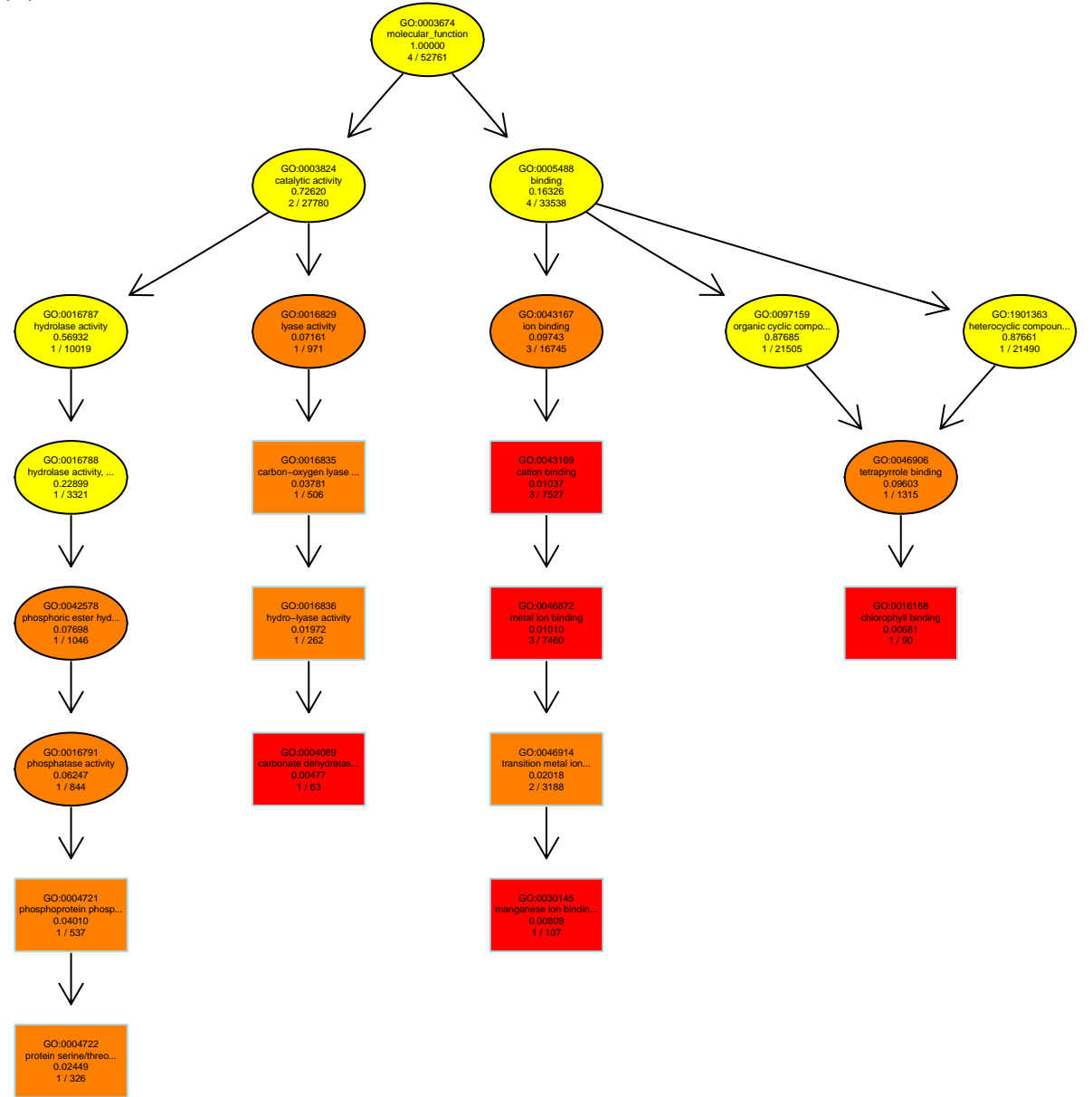

Supplement: Supplementary file 1 [file plants-12-02855-s001.zip › plants-2467175-supplementary/Supplementary materials/Supplementary Files/Supplementary File 7.pdf]

(A)

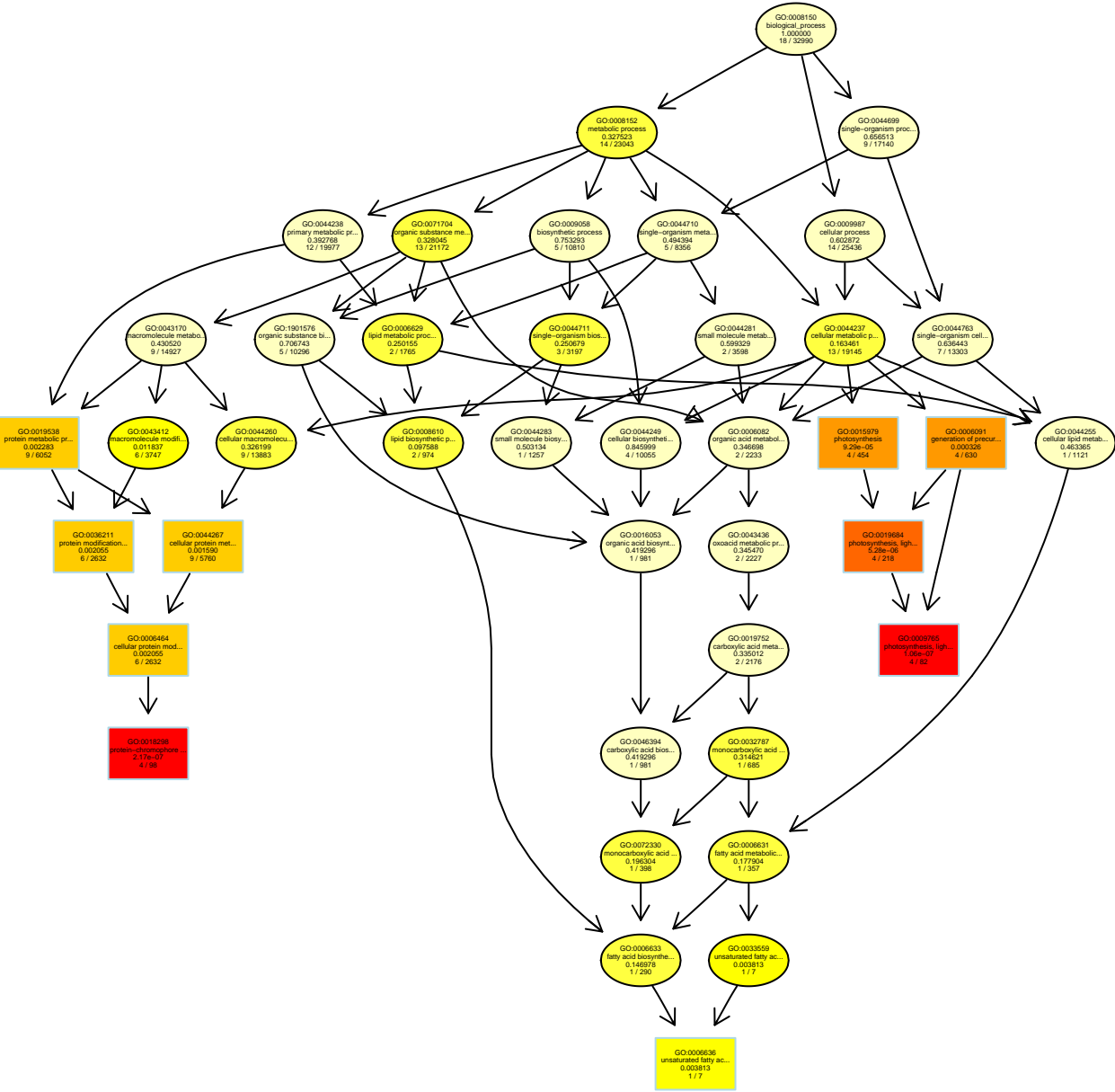

(B)

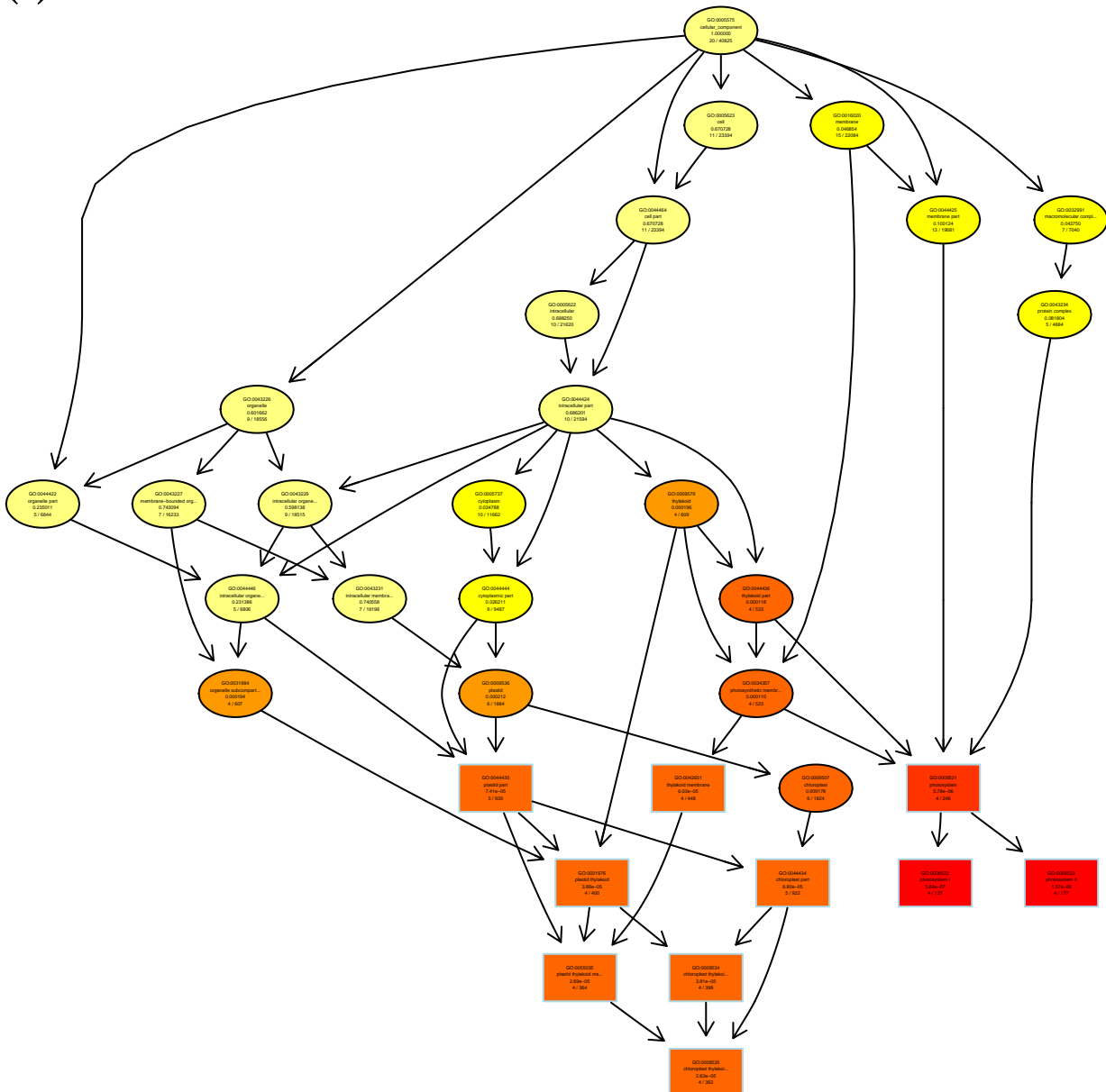

(C)

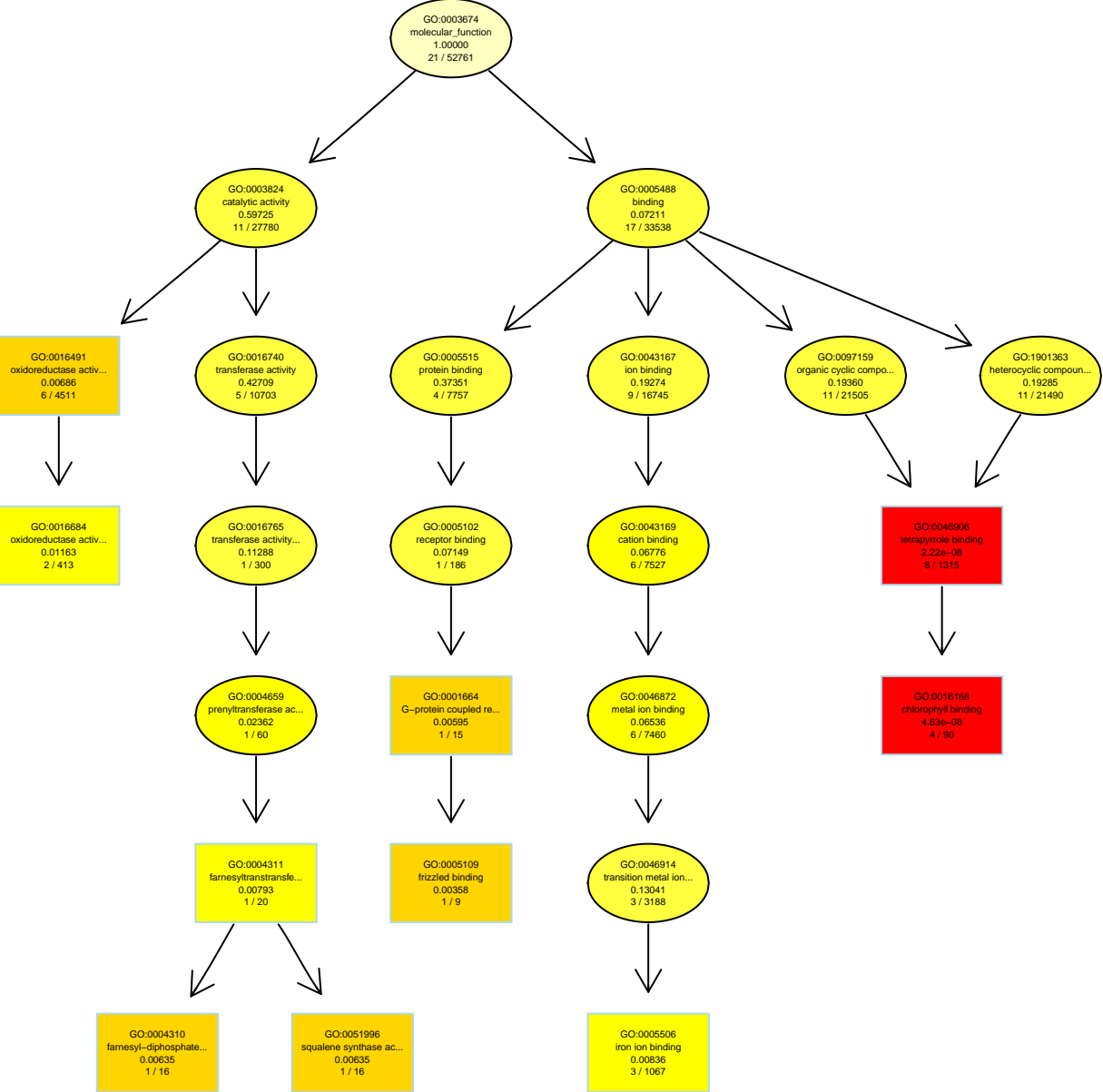

Supplement: Supplementary file 1 [file plants-12-02855-s001.zip › plants-2467175-supplementary/Supplementary materials/Supplementary Files/Supplementary File 8.pdf]

(A)

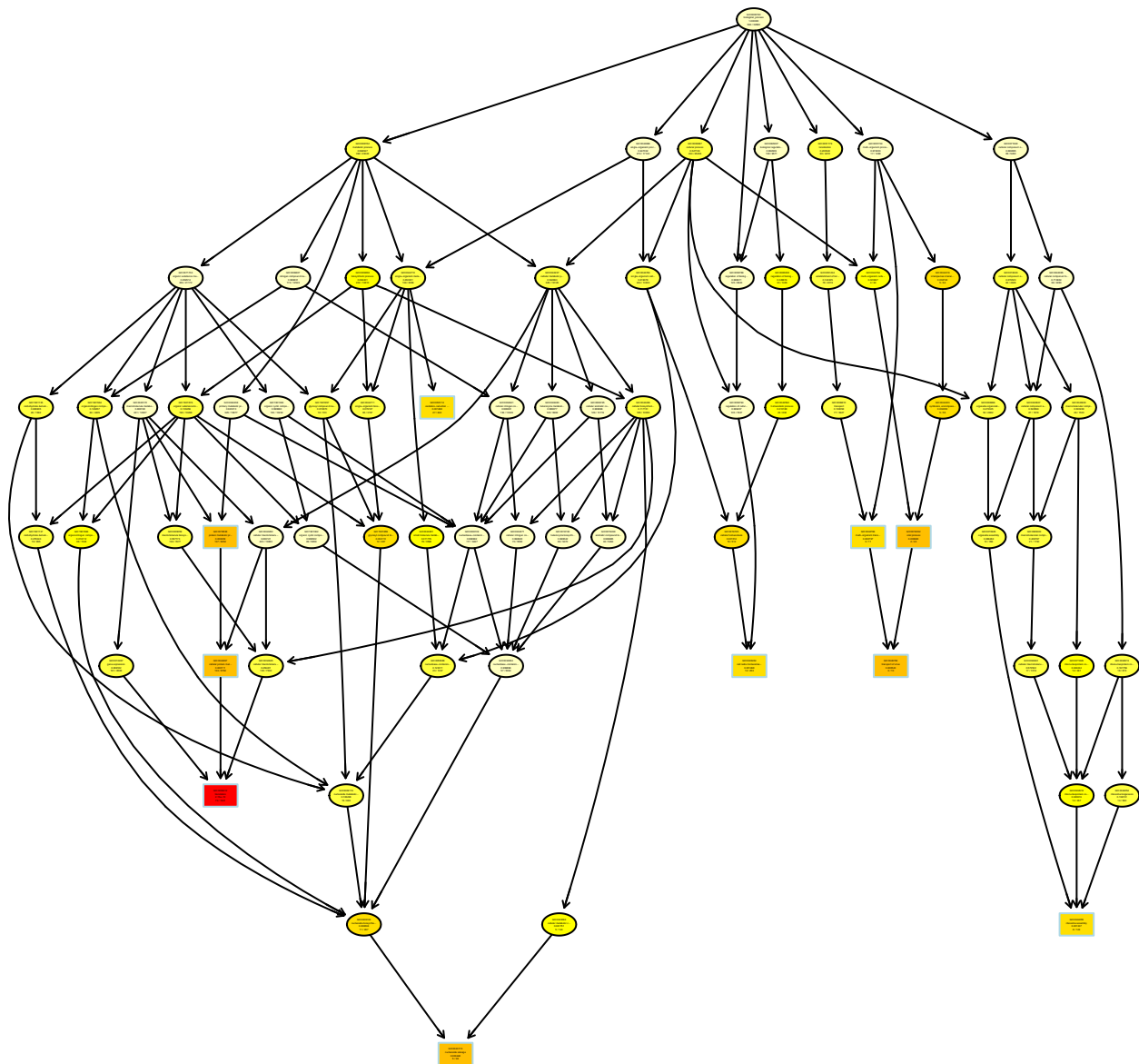

(B)

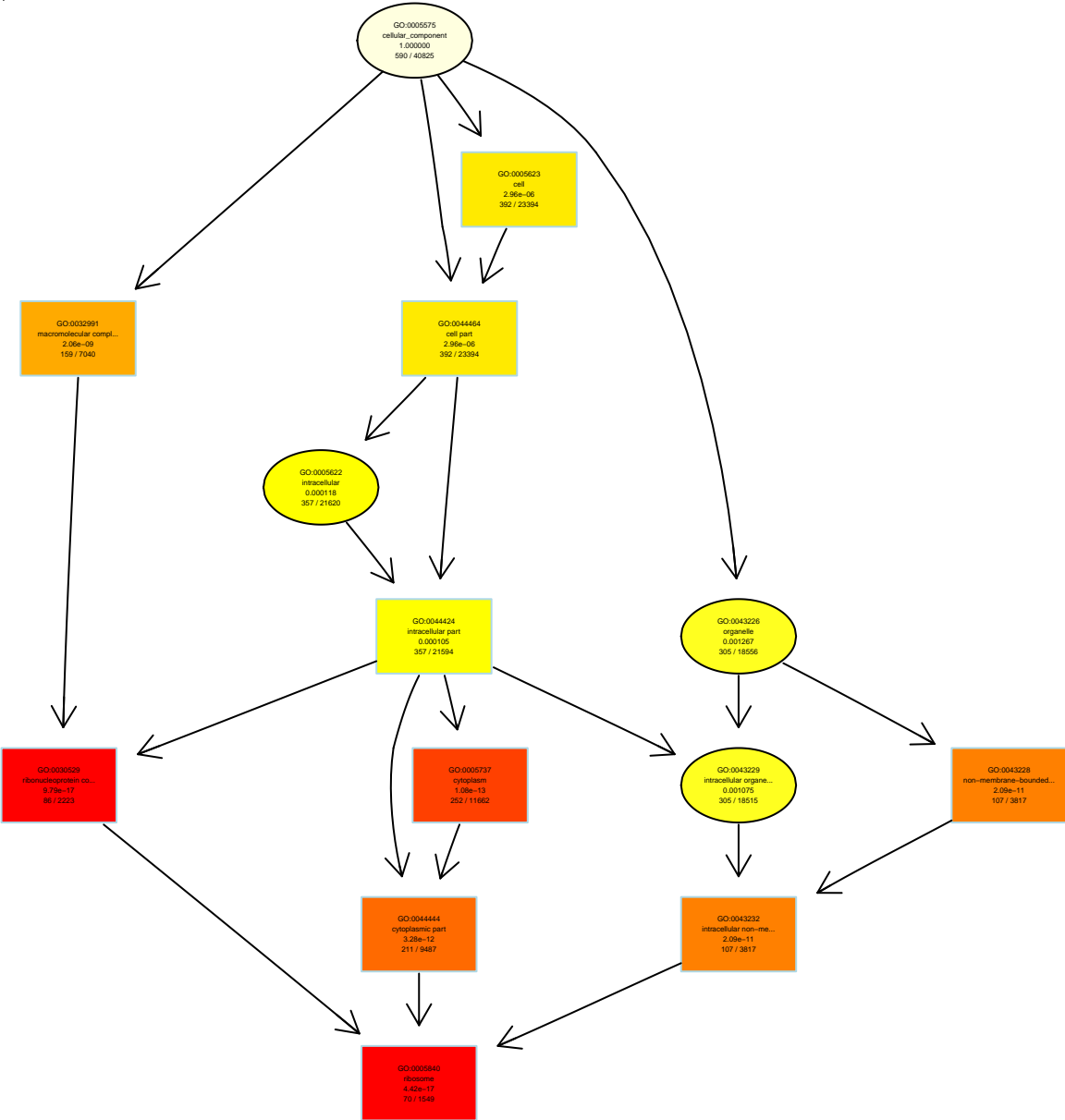

(C)

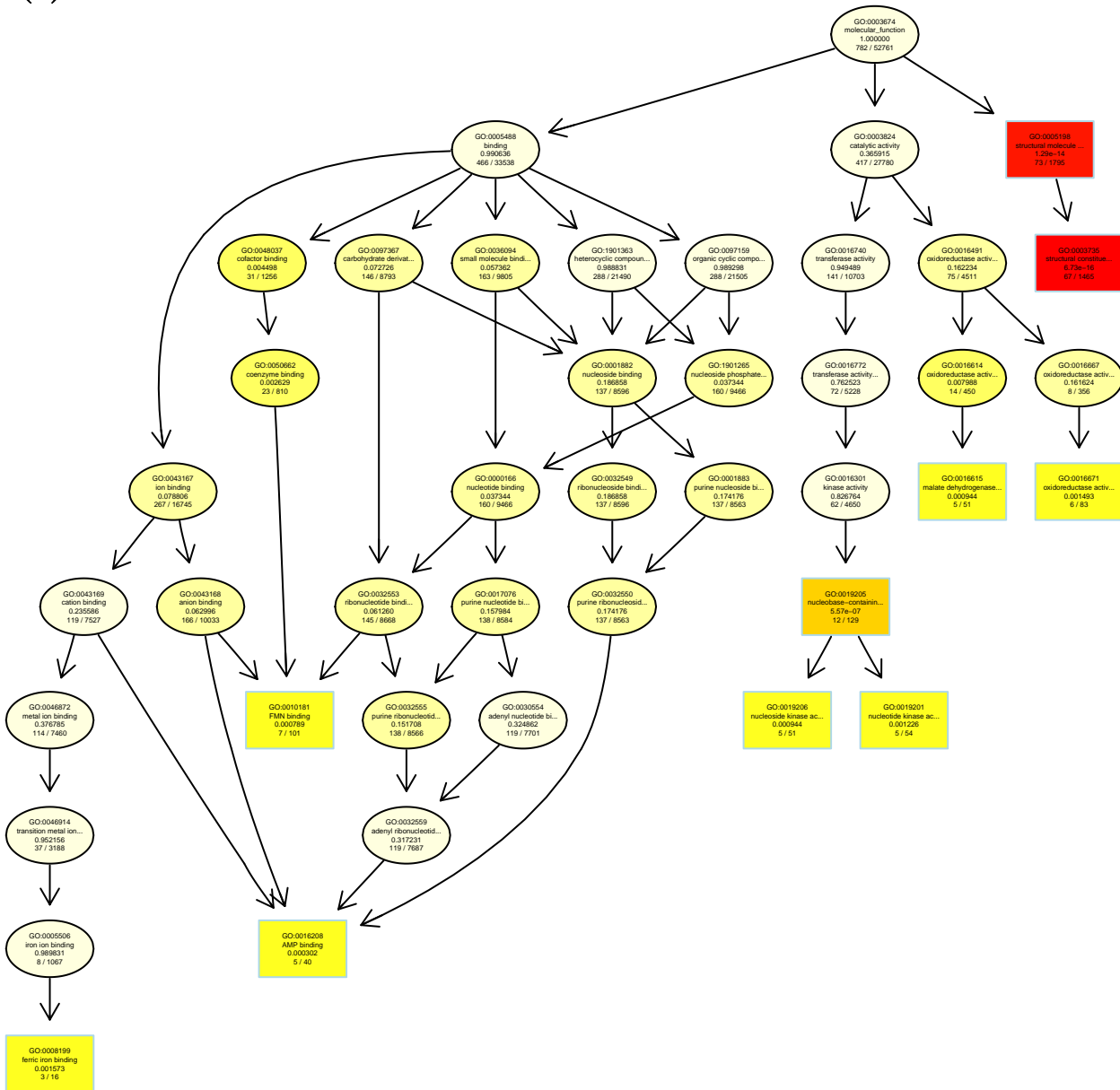

Supplement: Supplementary file 1 [file plants-12-02855-s001.zip › plants-2467175-supplementary/Supplementary materials/Supplementary Files/Supplementary File 9.pdf]
